# Redox-Controlled Chalcogen Bonding as a Modulator of ZnCl_2_ Chelation and Transport

**DOI:** 10.1021/jacs.5c19650

**Published:** 2026-02-24

**Authors:** You Jiang, François P. Gabbaï

**Affiliations:** Department of Chemistry, 14736 Texas A&M University, College Station, Texas 77843-3255, United States

## Abstract

Zinc is a ubiquitous metal in biological systems where an elaborate array of zinc transporters is involved in maintaining its homeostatic levels. Because toggling these levels may define new therapeutic approaches, strategies for the selective transport of Zn^2+^ ions are becoming increasingly coveted. Here, we describe a unique stimulus-responsive Zn^2+^ transporter, the activity of which can be adjusted by a redox-controlled intramolecular chalcogen bonding motif positioned at the heart of the construct. This system features a dipicolylamine (DPA) zinc chelator engaged, through its amino group, in a N–Te chalcogen bond with an adjacent diaryl tellurium moiety. Our work shows that oxidation of the tellurium center to the tetravalent state decreases the Zn^2+^ affinity of the DPA unit because of strengthened N–Te chalcogen bonding. We exploited this property for the differentiated transport of Zn^2+^ ions across phospholipid bilayers while also demonstrating the stimulus-responsive nature of this system in a set of experiments where transport is initiated *in situ* through reduction of the Te^IV^ form of the transporter into its divalent counterpart using glutathione.

## Graphical abstract

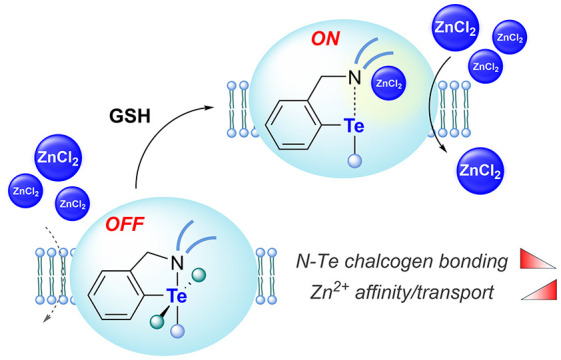

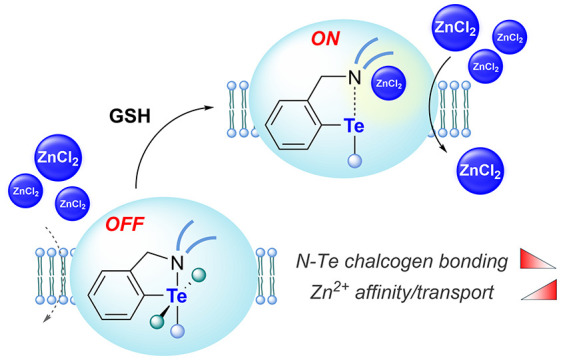

## Introduction

Zinc is a crucial trace metal for cellular processes,^1^ controlling the expression and activation of biological molecules such as transcription factors^2^ and enzymes.^3^ Given the essential role that this metal ion plays, being able to perturb its concentration or availability at the cellular level may be leveraged to further interrogate its role in cellular processes^4^ while also opening the door to possible therapeutic applications.^5^ As a small divalent, highly solvated ion, its intentional trafficking, particularly across phospholipid bilayers, is also a fundamentally stimulating objective that has begun to attract attention.^6^ These fundamental and applied considerations have motivated several efforts aimed at the design of artificial Zn^2+^ transporters.^7^ Some of the simplest motifs explored to date include PBT2 (**A**, Figure 1), a quinoline derivative whose antibacterial properties have been associated with its Zn^2+^ transport activity.^8^ Another simple Zn^2+^ ionophore is pyrithione (**B**, Figure 1) used to treat various dermatological conditions.^9^ Recent developments in this area of chemistry have explored the potential of a strategy based on classical Zn^2+^ chelators.^10^ This strategy is exemplified in the work of Choudhary, who utilized the well-known dipicolylamine (DPA) motif^11^ and proposed that the Zn^2+^ transport properties of such compounds can be adjusted through steric tuning of the binding pocket using peripherally installed silyl substituents as in the case of **C** (Figure 1).^10a^ With these prior findings as a backdrop and as part of our ongoing interest^12^ in the design of stimulus-responsive ion transporters,^13^ we have recently questioned whether such DPA-based systems could be engineered into platforms that can be activated *via* redox chemistry.

**Figure 1:**
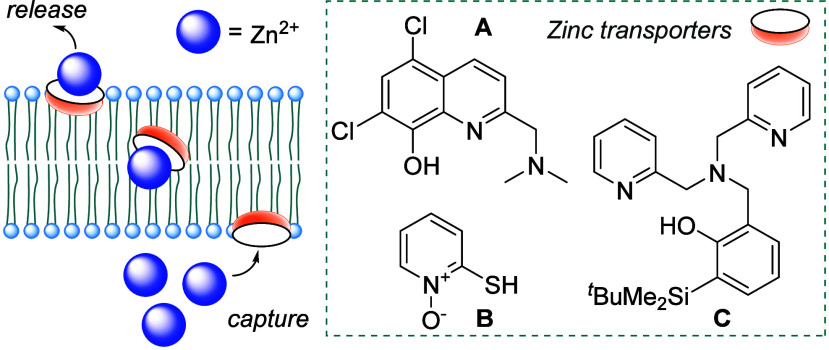
Zinc transport across a phospholipid bilayer and examples of small-molecule zinc transporters.

Our search for an effective redox trigger that could be incorporated into such an architecture led us to consider the competitive engagement of the central amino group of the DPA unit into a chalcogen bond (Figure 2) as a means to modulate the Zn^2+^ affinity of the tridentate chelator. Chalcogen bonds,^14^ which arise when a Lewis base engages in secondary electrostatic and charge transfer interactions with a group 16 atom, are present in simple derivatives such as **D** (Figure 2). They also get significantly stronger upon oxidation of the chalcogen atom, as illustrated by the N–Te bond shortening of over 0.4 Å observed upon conversion of **D** into **E** (Figure 2).^15^ The strengthening of this interaction can be assigned to an increase of surface electrostatic potential (*V*_*s,max*_) defining the tellurium-based σ-hole and a decrease in the energy of the corresponding accepting σ* orbital, as illustrated in Figure 2. Returning to our original intent to encode redox modulation within our target construct, we hypothesized that the variable strength of the N–Te chalcogen bond in such derivatives would provide a handle over the Zn^2+^ affinity of a DPA chelator, provided that the DPA analogs of compounds of type **I** and **II** could be assembled (Figure 2). In addition to redox-controlled binding,^16^ we also envisioned that the transport properties of these systems could be similarly affected, providing opportunities for stimulus-responsive transport. Our results are presented herein.

**Figure 2:**
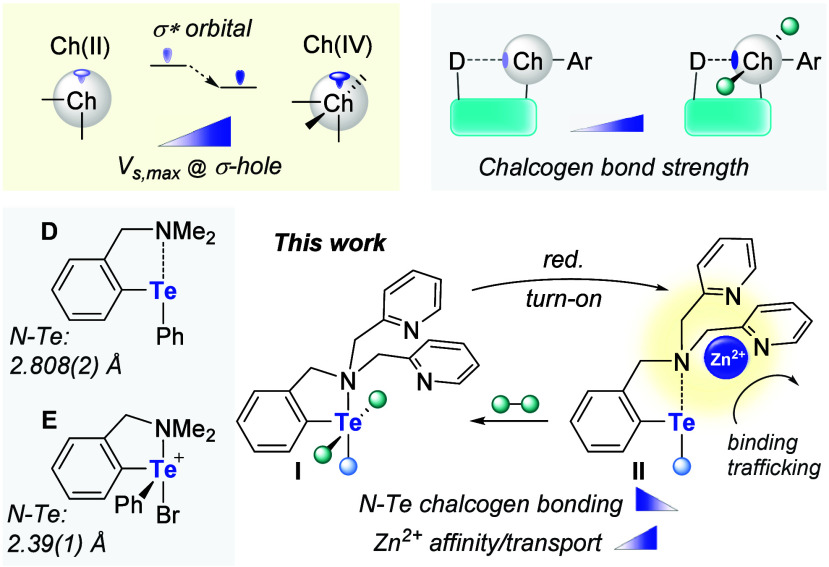
Top: Orbital and electrostatic origin of chalcogen bonding interactions, along with the effect of chalcogen oxidation. The strengthening of the interaction induced by oxidation of the chalcogen is also depicted. Bottom: Experimental precedent for the oxidation-induced strengthening of chalcogen bonding in the case of an intramolecular tellurium ammino motif and objective of the current study.

## Results and Discussion

### Synthesis and Characterization

As shown in Figure 3, assembly of the target compound began with the generation of Ar^F^TeBr (**a**, Ar^F^ = 3,5-C_6_H_3_(CF_3_)_2_), *in situ* from the corresponding ditelluride and Br_2_, followed by reaction with a freshly prepared solution of the Grignard reagent **b**. Under acidic conditions, the resulting asymmetrical telluride **c** was converted into its aldehyde counterpart **d**. Using inspiration from related protocols in the literature,^17^ this aldehyde was reduced with excess NaBH_4_ and then brominated using PBr_3_ to afford **e**. Compound **e** was allowed to react with dipicolylamine under basic conditions to produce the divalent tellurium derivative **1** as an off-white solid. This compound has been fully characterized. Its ^1^H NMR spectrum (Figure S17) features a set of well-resolved resonances congruent with the expected structure. Two resonances, that appear in a 2:1 ratio at 8.25 and 7.81 ppm, are readily assigned to the Ar^F^ moiety. The structure of this new derivative has been confirmed using single-crystal X-ray crystallography (Figure 3). The most conspicuous feature concerns the formation of a short interaction of 2.842(2) Å between the tertiary nitrogen atom of the DPA moiety and the tellurium center. The Lewis basic nitrogen sits directly trans from the Ar^F^, suggesting the formation of a σ-hole interaction or, in this precise case, a chalcogen bond.^14d^ The length of this chalcogen bond is comparable to that found in other compounds,^18^ including **D** (2.808(2) Å).^15a^ The structure of this derivative has been computationally studied using Density Functional Theory (B3LYP-D3­(BJ) functional; basis set: def2-TZVP). As indicated by the computed N–Te distance at 2.913 Å, the optimized structure of **1** is in good agreement with that experimentally determined, pointing to the suitability of the level of theory chosen for this study. The presence of a chalcogen bond is supported by a Natural Bond Orbital (NBO) analysis as illustrated in Figure 3, which identifies a lp­(N)→σ*­(Te–C) donor–acceptor interaction that contributes 6.7 kcal/mol to the stability of the molecule at the second-order of perturbation theory. This stabilization energy is comparable to that computed for other N/Te systems featuring N→Te^II^ interactions.^19^

**Figure 3:**
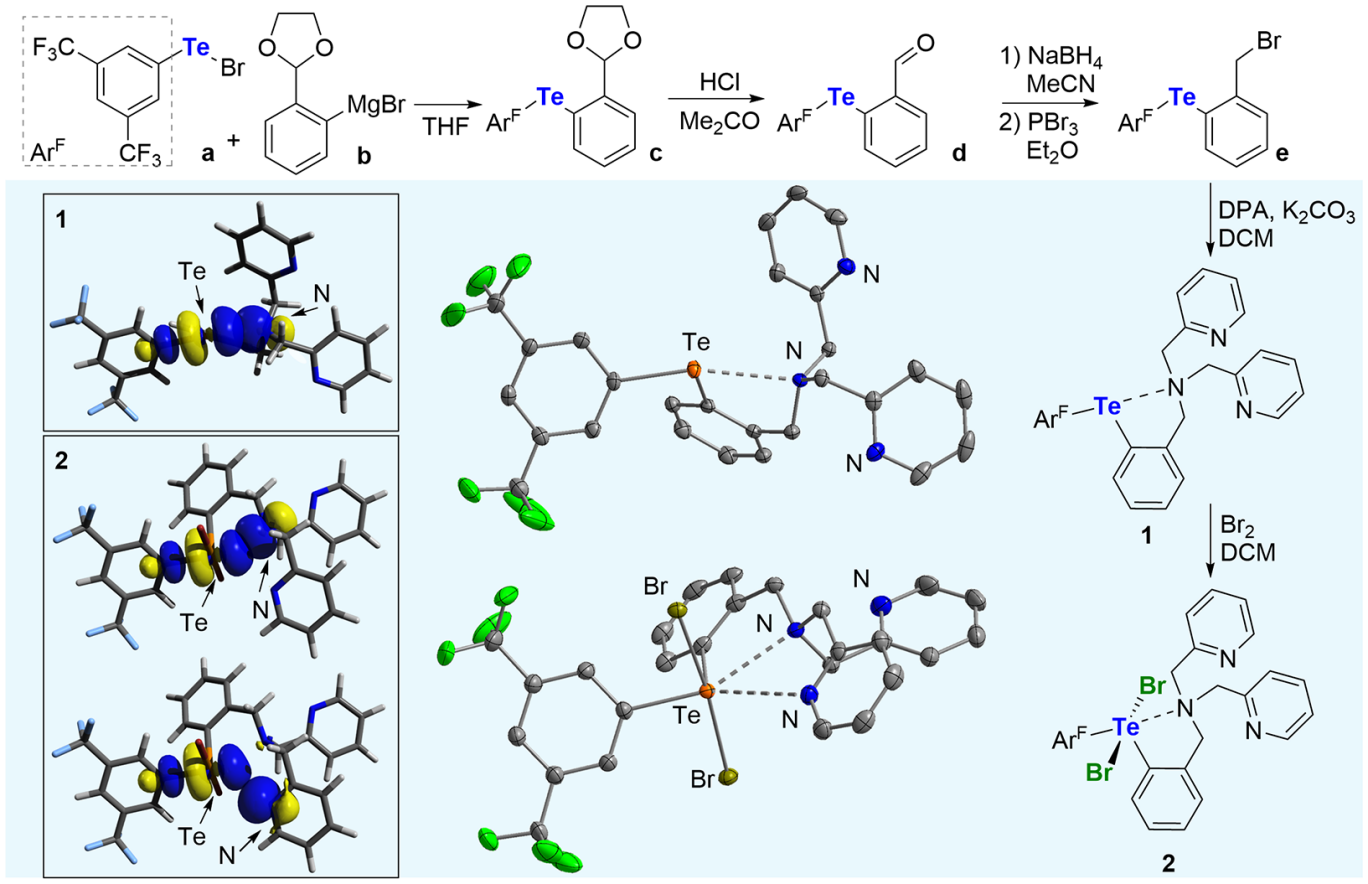
Synthesis and solid-state structures of **1** and **2**, drawn at the 50% probability level. The insets show the main NBOs associated with the N–Te interactions in each compound (isovalue = 0.05).

Given our intent to use the redox state of tellurium as a handle over the properties of such constructs, oxidation of the tellurium center became the next objective. Using conditions previously used for the oxidation of diaryl tellurides,^20^ **1** was treated with one equivalent of Br_2,_ which resulted in the clean formation of the tetravalent tellurium dibromide derivative **2**, as shown in Figure 3. This derivative, which was isolated as a yellow solid, can be handled in air like its divalent precursor. Its ^1^H NMR spectrum (Figure S21) shows a downfield shift of the Ar^F^ resonances which appear at 8.84 and 8.00 ppm instead of 8.25 and 7.81 ppm in **1**. The ^125^Te NMR (Figure S24) shows a single resonance at 962.7 ppm, which is comparable to that measured for other Te^IV^ compounds with intramolecular coordinating N-donor substituents,^18^ notably downfield from that of **1** (683.7 ppm, Figure S20). The impact of tellurium oxidation on the N–Te chalcogen bond was assessed through a determination of the structure of **2** by single-crystal X-ray diffraction (Figure 3). This analysis confirmed the oxidation of the chalcogen into a tellurium dibromide moiety, with the two halides positioned trans from one another. This analysis also indicates a shortening of the N–Te distance from 2.842(2) Å to 2.738(3) Å in **2**, which is comparable to that found in similar telluroxides.^21^ Further inspection of the structure revealed the presence of an additional N–Te contact of 3.336(3) Å involving one of the pyridyl rings. The strengthening of the primary N–Te donor–acceptor interaction can be assigned to a lowering of σ*­(Te–C) orbital and a deepening of the associated σ-hole, induced by oxidation. We have previously discussed such effects upon oxidation of tellurides into telluronium cations.^22^ Even if the bond shortening is modest, we speculated that its impact on the stability of the linkage may be substantial. Indeed, an NBO analysis displayed in Figure 3 shows that the lp­(N)→σ*­(Te–C) donor–acceptor interaction energy (E(2) = 10.2 kcal/mol) increases by 3.5 kcal/mol when compared to that in **1**. Even if this energy difference appears small, we should bear in mind that it could have a substantial effect on the position of any equilibrium involving dissociation of the N–Te bond.^23^ Moreover, the NBO analysis also identifies a second lp­(N)→σ*­(Te–C) interaction involving the nitrogen atom of one of the pyridyl moieties. Although weaker, this additional interaction associated with E(2) = 2.7 kcal/mol indicates that the pyridyl donors may also be recruited by the Lewis acidic tellurium center through chalcogen bonding. These additional interactions made us question whether the DPA unit of **2** would be available for Zn^2+^ binding.

### Zinc Coordination Study

To assess the impact of N–Te chalcogen bonding on the affinity of these derivatives for Zn^2+^ ions, both **1** and **2** were titrated with ZnSO_4_ in 1:1 MeOH/DMSO. UV–vis monitoring of these titrations (Figures S32 and S33) showed notable changes in the corresponding absorption spectra, confirming that both derivatives bind Zn^2+^. Fitting the data to a 1:1 binding isotherm^24^ indicates that the divalent derivative **1** displays a Zn^2+^ binding constant of 5.4 ± 1.0 × 10^4^ M^–1^, which is almost three times larger than the value of 1.9 ± 0.5 × 10^4^ M^–1^ measured for **2** (Figure 4). These results are consistent with the increasingly competitive engagement of the DPA moiety of **2** in a chalcogen bonding interaction. These results also validate our hypothesis that N–Te chalcogen bonding allows for effective modulation of the affinity of the DPA moiety for the divalent ion, which is notably decreased in the tetravalent derivative. This conclusion is supported by an *in silico* analysis of the differential ZnCl_2_ affinity of **1** and **2**. Indeed, the computed ΔG of the isodesmic reaction (Table S5) in eq 1 affords a differential binding constant at room temperature of 4.8, close to the experimental value of 2.8, obtained from the ratio of the aforementioned binding constants.

1 $$1+2{\mathrm{‐ZnCl}}_{2}⇌2+1{\mathrm{‐ZnCl}}_{2},\Delta G=-3.9\;\mathrm{kJ}/\mathrm{mol}$$

**Figure 4:**
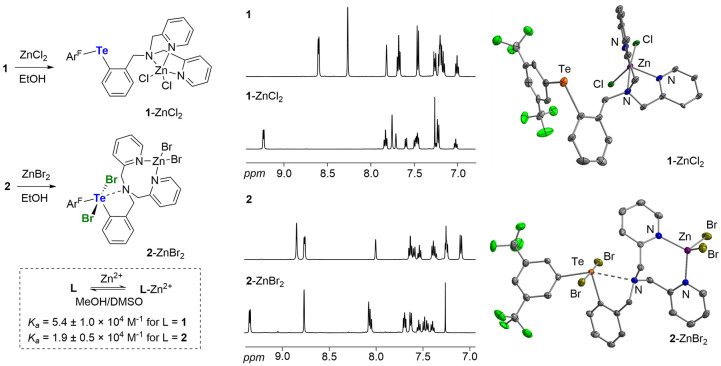
Left: Synthesis of zinc coordinated complexes and experimental binding constants of **1** and **2** with Zn^2+^ in 1:1 MeOH/DMSO. Middle: Comparisons of the ^1^H NMR spectra measured for **1**, **2** and their corresponding Zn^2+^ complexes **1**-ZnCl_2_ and **2**-ZnBr_2_. Right: Solid state structures of **1**-ZnCl_2_ and **2**-ZnBr_2_, drawn at the 50% probability.

To support these UV–vis spectroscopic studies, we also attempted to observe the formation of Zn^2+^ complexes by NMR spectroscopy in CDCl_3_, which is illustrated in Figure 4. Formation of these complexes manifests in notable changes in the ^1^H NMR spectra of the receptors, including a downfield shift of the CH unit at the 6-position of the picolyl moiety. The signals of the Ar^F^ substituent are also affected, especially in the case of the divalent system, which shows an upfield shift of the *ortho*-proton resonances from 8.25 ppm in **1** to 7.75 ppm in **1**-ZnCl_2_. Also, the ^125^Te NMR chemical shift changes from 683.7 ppm in **1** to 652.6 ppm in **1**-ZnCl_2_. The latter is close to the value measured for intermediate **e** (639.4 ppm), suggesting displacement of the chalcogen bond upon zinc binding. Single crystals of **1**-ZnCl_2_ and **2**-ZnBr_2_ could be grown by diffusion of pentane into an equimolar mixture of the receptor and the zinc dihalide in chloroform (Figure 4). These structures confirm the differing coordination mode of the DPA ligand to the zinc atom. In the case of **1**-ZnCl_2_, coordination of the zinc ion occurs at the expense of the weak N–Te interaction present in **1**, as supported by the N–Te distance which increases from 2.842(2) Å in **1** to 4.40(1) Å in **1**-ZnCl_2_. Conversion of **2** into **2**-ZnBr_2_ induces an elongation of the N–Te distance from 2.738(3) Å to 2.931(3) Å, suggesting weakening of the chalcogen bond rather than complete displacement. The retention of this chalcogen bonding interaction, even in the presence of a Zn^2+^ ion coordinated to the DPA ligand, illustrates how its strength is favorably impacted by the high valent nature of the tellurium center. It is worth noting that, in **2**-ZnBr_2_, the zinc ion is only coordinated through the pyridyl nitrogen atoms, leading to a rare eight-member chelate ring.^25^ Formation of this unusual motif serves as a reminder that the benzylic amino group remains engaged in a chalcogen bond with the Te^IV^ center.

### Transport Study

To test the Zn^2+^ transport properties of these systems, we decided to use a recently published assay based on the use of Magnesium Green (MgG) as a Zn^2+^ responsive probe loaded in the interior of large unilamellar vesicles (LUVs) formulated using egg-yolk phosphatidylcholine (EYPC, 80%) as the lipid source, combined with cholesterol (20%) for increased stability.^10b,10c^ These LUVs were subsequently treated with the transporter (0.1 mol% based on lipid concentration) and allowed to mature for 2 min to ensure adequate recruitment of the transporters into the liposomal membranes. At the conclusion of this maturation time, which is defined as *t* = 0, the MgG fluorescence was monitored for 30 s to verify the stability of the system. Transport was then initiated by induction of a 1 mM ZnCl_2_ gradient. MgG fluorescence was recorded for an additional 420 s before lysis of the vesicles using Triton-X. The resulting data is shown in Figure 5. These experiments reveal a marked difference between the activity of compounds **1** and **2**. Under the conditions of this assay, compound **1** is particularly potent, reaching a plateau after just 2 min. Comparably, **2** displays a much lower transport activity, as indicated by the slow rise of the fluorescent signal, which reaches only a normalized intensity (*I*_*N*_) value of 6% vs 50% in the case of **1**. The contrasting behavior of these two compounds is also manifested in their *EC*_50_, which could be readily derived from a classical Hill analysis (Figures S37 and S38). Indeed, carrying out this analysis using data collected at *t* = 400 s affords *EC*_50_ for **1** (9.2 × 10^–3^ mol% based on lipid concentration) and **2** (158.6 × 10^–3^ mol% based on lipid concentration), showing a greater than one-order of magnitude differential in the activity of these two compounds. The higher activity of **1** is also supported by an MgG assay in which the two forms of the systems, namely **1** and **2**, were preincorporated in the membrane (Figure S42). We propose that the lower activity of **2** results from the strength of the N–Te^IV^ chalcogen bonding which challenges effective ZnCl_2_ complexation by the DPA chelator, affecting the capture and thus transport of the cargo.^26^ The picture that emerges from these transport studies is one in which adjusting the involvement of the tertiary nitrogen atom of the DPA ligand in a chalcogen bonding interaction can be used to control transport.

**Figure 5:**
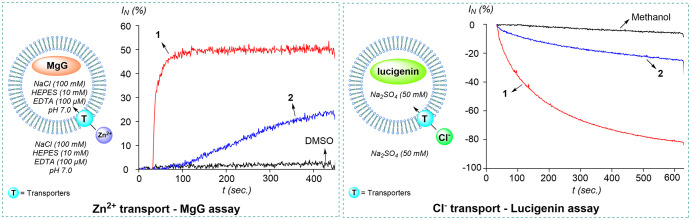
Left: Schematic representation of the MgG assay and resulting Zn^2+^ influx traces elicited by addition of a DMSO solution of transporters **1** and **2** (0.1 mol% with respect to lipids). Right: Schematic representation of the lucigenin assay and influx traces elicited by addition of a methanol solution of transporters **1** and **2** (1 mol% with respect to lipids). The MgG and lucigenin assays are carried out using EYPC/cholesterol (8:2) and POPC/cholesterol (7:3) LUVs, respectively.

The transport of Zn^2+^ ions alone would generate an unlikely charge gradient if not balanced by chloride influx. To verify that chloride transport also occurs, we complemented the above metal-responsive MgG assay with a chloride-responsive one based on lucigenin.^27^ The latter was loaded into 7:3 POPC (1-palmitoyl-2-oleoyl-*sn*-glycero-3-phosphocholine) /cholesterol vesicles that were subsequently treated with the transporter (1 mol% based on lipid concentration). After a 2 min maturation period, considered to ensure proper recruitment of the transporter into the liposomal membranes, transport was initiated by inducing a 12.5 mM transmembrane gradient through administration of a ZnCl_2_ solution. Chloride influx was followed by monitoring the lucigenin fluorescence intensity for a period of 10 min. Each experiment was concluded by lysis of the vesicles using the detergent Triton-X (Figure 5). These experiments reveal a marked difference between the activity of the two derivatives. Indeed, while **1** appears particularly potent, eliciting 81% influx at the end of the assay, compound **2** shows significantly lower activity. These results mirror the trend observed in Zn^2+^ transport, indicating that zinc cations and chloride anions are co-transported. It follows that **1** can be described as an active ZnCl_2_ transporter.

Given the contrasting transport properties of **1** and **2**, we decided to test whether the *in situ* reduction of **2** could serve to elicit transport. This possibility was reinforced by a series of studies that have explored chalcogen reduction as a means to trigger transport, using glutathione (GSH), a biologically relevant reductant,^12,28^ or dithiothreitol (DTT), an electron-rich thiol often used in biophysical studies.^28c,29^ A particularly relevant example is that recently provided by the group of Langton, who found that reduction of Ar^F^_2_TeO_2_ with DTT affords the corresponding telluroxide Ar^F^_2_TeO as an efficient Cl^–^/OH^–^ antiporter.^29^ Sulfonium ion reduction by GSH as a way to influence transport provides another pertinent precedent for this reduction-induced turn-on strategy.^12^ Building on these precedents, we decided to test whether transporter **2** could also be reduced into its more active counterpart **1**. Toward this end, we first used ^1^H NMR spectroscopy to investigate the reduction of **2** using GSH. Combining this Te^IV^ compound with GSH in DMSO-*d*_6_, resulted in the rapid formation of **1** as illustrated by the spectra in Figure 6. This result, which indicates efficient reduction of the tetravalent tellurium center, also suggests that GSH could be used to elicit transport, provided that reduction of **2** can also be carried out *in situ*, during the vesicle assay. With this in mind, we moved to investigate the possibility of stimulus-responsive transport across liposomal membranes through the *in situ* reduction of **2** into **1**. This scenario was tested with MgG-loaded vesicles pulsed with ZnCl_2_ as described above. At *t* = 100 s, 10 equiv. of GSH (1 mol% based on lipid concentration) was added to the medium. Inspection of the MgG fluorescence signal showed that this addition induced an accelerated influx of Zn^2+^ ions (Figure 7). The extent of acceleration can be correlated to the amount of GSH employed. Indeed, adding simply 3 equiv. of GSH leads to a weaker but still notable increase in Zn^2+^ influx (Figure S40), strengthening our argument that *in situ* reduction of **2** into **1** is responsible for the enhanced transport. Realizing the speed at which **2** is reduced by GSH influences the observed rise in transport activity, we carried out an additional assay in which **2** and GSH (3 equiv.) were first added to the liposomal solution, which was allowed to mature for 2.5 min prior to administration of a zinc pulse. The resulting Zn^2+^ influx (Figures 7 and S41), measured in this experiment, was almost superimposable with that obtained with pure **1**, providing further validation for our hypothesis of reduction-induced turn-on transport. The favorable influence of GSH on Zn^2+^ transport was also verified using vesicles in which **2** was preincoporated in the membrane (Figure S42). We have also verified the effect of GSH in the chloride-responsive lucigenin assay. Given that **2** shows negligible activity in this POPC-based assay, an even more apparent turn-on effect was observed, as illustrated in Figure 7, where GSH (3 mol% based on lipid concentration) was added to the medium 3 min after the ZnCl_2_ pulse, resulting in the accelerated decrease of the lucigenin fluorescence intensity.

**Figure 6:**
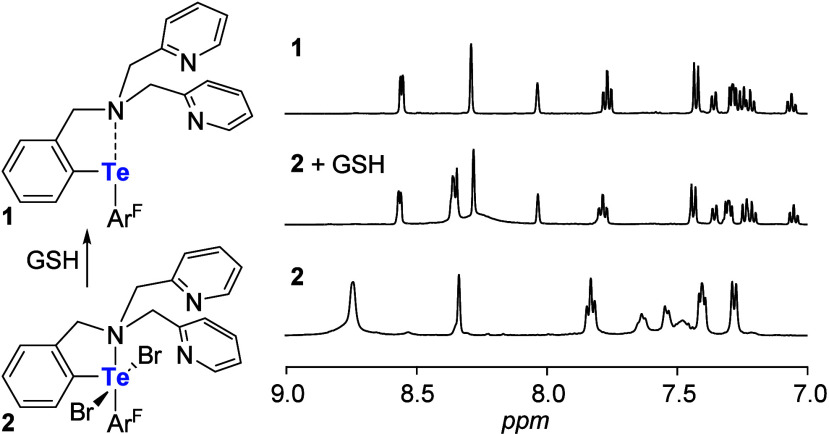
Reduction of **2** into **1** using GSH in DMSO-*d*_6_ and corresponding partial ^1^H NMR spectra showing the reaction progress. The spectrum of **2** was recorded at 50 °C. The reduction was carried out at 25 °C and recorded at the same temperature. The top spectrum is that of pure **1** at 25 °C.

**Figure 7:**
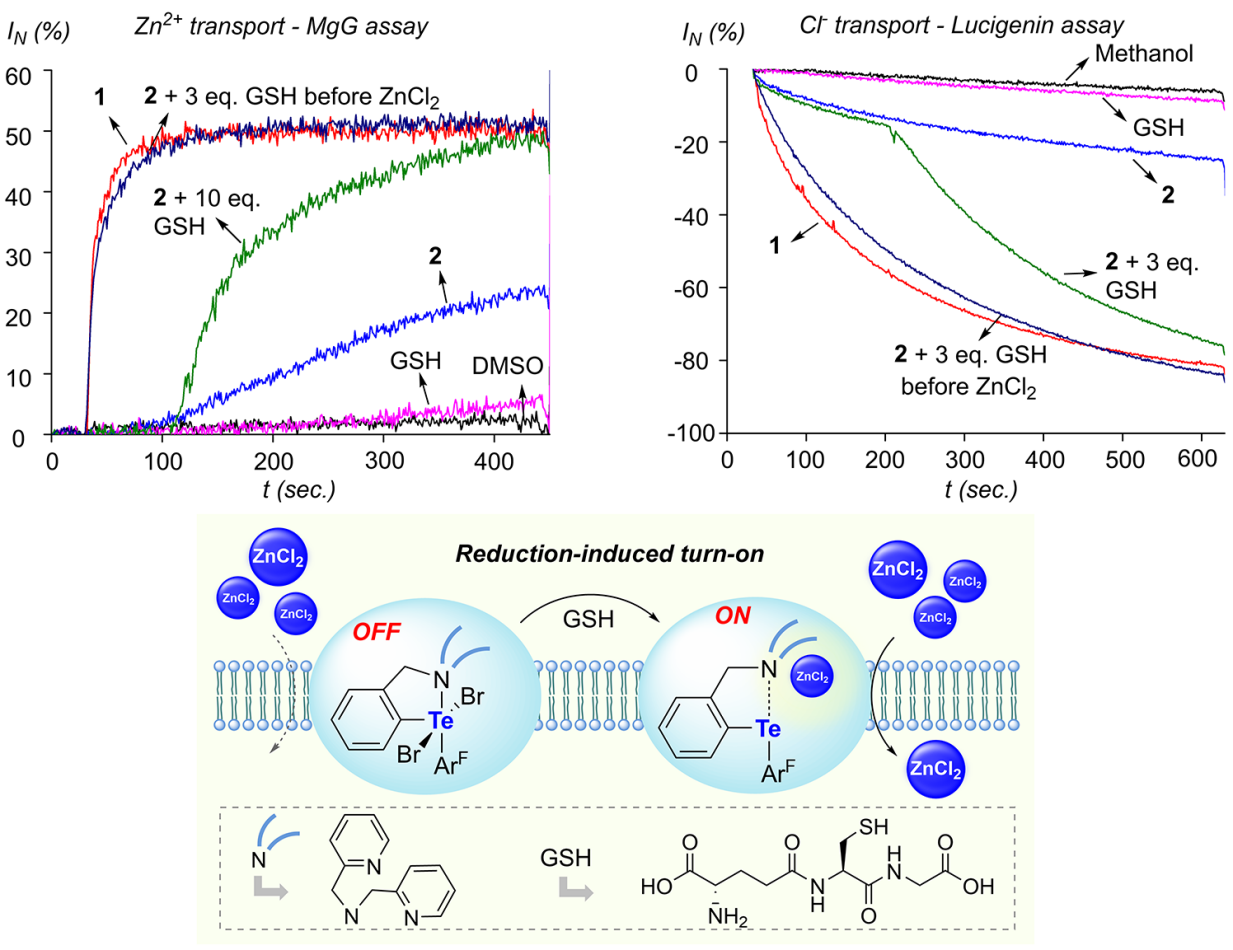
Top left: MgG assay results showing the Zn^2+^ influx traces obtained upon *in situ* activation of **2** (0.1 mol% with respect to lipids) using GSH. The influx profile obtained with pure **1** (0.1 mol% with respect to lipids), and the background transport observed with GSH and DMSO are also included for comparison. Top right: Lucigenin assay results showing the Cl^–^ influx profiles obtained upon *in situ* activation of **2** (1 mol% with respect to lipids) using GSH. The influx profile obtained with pure **1** (1 mol% with respect to lipids), and the background transport observed with GSH and MeOH are also included for comparison. Bottom: Illustration of the GSH-induced activation of transport.

## Conclusion

This work establishes a new concept that leverages the competitive engagement of nitrogen-based metal chelators into intramolecular chalcogen bonding motifs. Owing to the presence of this intramolecular interaction, the affinity of the chelator for the metal is decreased, providing a handle not only over metal binding but also over its transport across biological membrane mimics. This phenomenon is demonstrated here using divalent zinc, a metal cation of great importance in biology, and tellurium, a privileged element for strong chalcogen bonding interactions. An essential aspect of this research is the demonstration that the redox state of the tellurium involved can be used to modulate the strength of the intramolecular chalcogen bond and thus the transport activity of the constructs. This strategy enables access to unique transporters whose function is directly regulated by the redox state of the chalcogen atom, providing a mechanism for *in situ* turn-on of transport properties. Although demonstrated here specifically with zinc, the approach should be broadly applicable to other metal-specific chelators, thereby broadening the impact of these findings. We are currently engaged in these explorations.

## Supplementary Material



## Notes

Synthetic details; recorded NMR and UV–vis spectra; transport study details and recorded ion influx profiles; X-ray diffraction details; computational details, thermochemical analyses, and optimized structures (PDF)
