# Supplementary material for: Redox-Controlled Chalcogen Bonding as a Modulator of ZnCl2 Chelation and Transport
Source: J Am Chem Soc. 2026 Feb 24;148(9):9540–7. doi: 10.1021/jacs.5c19650 (PMC12983301; doi:10.1021/jacs.5c19650)
Supplement: Supplementary file 1 [file ja5c19650_si_001.pdf]

Redox-controlled chalcogen bonding as a modulator of $ZnCl_2$ chelation and transport

You Jiang and François P. Gabbaï*

Department of Chemistry, Texas A&M University, College Station, TX 77843-3255, United States of America

email: francois@tamu.edu

Supporting Information

Contents

1 Synthesis and Characterization S2
1.1 General Considerations S2
1.2 Synthetic Procedures S2
1.3 NMR Spectra S7
2 Zinc Ion Binding Studies S23
3 Reduction Studies S25
4 Transport Studies S27
4.1 Vesicles Preparation S27
4.2 Zinc Ion Transport Activity Studies S27
4.3 Hill Analyses of the Zinc Transport Activity S28
4.4 Reduction-Responsive Transport Studies Using the MgG Assay S29
4.5 Pre-Incorporation Transport Studies Using the MgG Assay S31
4.6 Chloride Ion Transport Activity Studies S32
4.7 Reduction-Responsive Transport Studies Using the Lucigenin Assay S33
5 X-ray Crystallographic Data S35
6 Computational Studies S36
6.1 General Methods S36
6.2 Optimized Structures and Coordinates of the Tellurium Compounds S36
6.3 Energy Calculations S40
References S41

1 Synthesis and Characterization

1.1 General Considerations

All experiments were carried out in an ambient environment unless otherwise noted. Chemicals were purchased from commercial suppliers and used without further purification. Egg yolk phosphatidylcholine (EYPC) and 1-palmitoyl-2-oleoyl-sn-glycero-3-phosphocholine (POPC) were purchased from Avanti Polar Lipids. The Sephadex G-50 column was purchased from GE Healthcare – Life Sciences. Bis[3,5-bis(trifluoromethyl)phenyl] ditelluride was prepared according to literature procedures.[1] Diethyl ether (Et_2O) and tetrahydrofuran (THF) were dried by refluxing over Na/K. All other solvents were ACS reagent grade and used as received. ^{1}H NMR spectra were recorded at room temperature using a Varian Inova 500 FT NMR spectrometer, a Bruker Avance 500 NMR spectrometer, or a Bruker Ascend 400 NMR spectrometer. ^{13}C{^{1}H} NMR spectra were recorded at room temperature using a Bruker Avance 500 NMR spectrometer. ^{19}F and ^{125}Te{^{1}H} NMR spectra were recorded at room temperature using a Bruker Ascend 400 NMR spectrometer. Chemical shifts are given in ppm. ^{1}H and ^{13}C signals were referenced to residual ^{1}H or ^{13}C solvent signals. The ^{19}F signals were referenced using C_6F_6 as a secondary external standard set at -161.64 ppm *vs.* $CFCl_3$.[2] The ^{125}Te signals were referenced using Ph_2Te_2 as a secondary standard set at 422.0 ppm vs. Me_2Te.[3] Elemental analyses were performed by Atlantic Microlab (Norcross, GA).

1.2 Synthetic Procedures

Synthesis of compound **c**

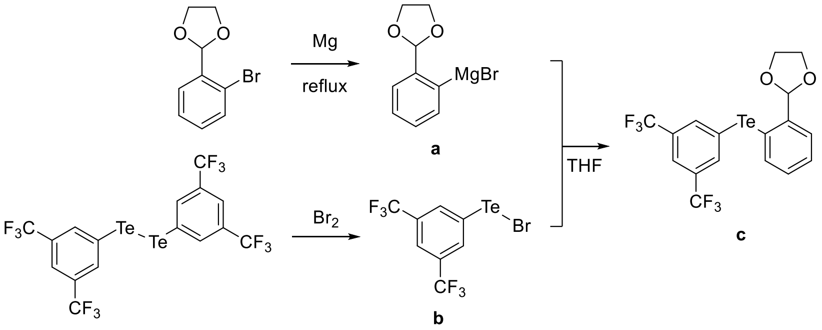

Under a nitrogen atmosphere, a solution of 2-(2-bromophenyl)-1,3-dioxolane (2.69 g, 11.74 mmol) in anhydrous THF (30 mL) was added dropwise to a Schlenk flask charged with magnesium beads (286 mg, 11.74 mmol) and a small crystal of I_2. The reaction mixture was heated at reflux for 1 h to afford a solution of compound **a**. In a separate Schlenk flask, bromine (0.30 mL, 938 mg, 5.87 mmol) was slowly added to a solution of bis[3,5-bis(trifluoromethyl)phenyl] ditelluride (4.00 g, 5.87 mmol) in anhydrous THF (30 mL). The resulting mixture was stirred for 30 min to afford compound **b**, which was transferred *via* cannula to the freshly prepared solution of compound **a**. The combined mixture was stirred overnight and then quenched with saturated aqueous NH_4Cl (40 mL).

The aqueous layer was extracted with Et_2O (3 × 40 mL). The combined organic phases were dried over $MgSO_4$, filtered, and brought to dryness under vacuum to afford a dark red residue. The crude mixture was purified by 100–200 mesh silica gel flash chromatography (Hexanes/CH_2Cl_2 = 7:3 as eluents) to give compound **c** as a yellow oil (3.70 g, 7.55 mmol, 64% yield). **^{1}H NMR** (500 MHz, $CDCl_3$) δ 8.23 (s, 2H, Ar-*H*), 7.83 (s, 1H, Ar-*H*), 7.55 (dd, *J* = 7.7, 1.6 Hz, 1H, Ar-*H*), 7.35–7.27 (m, 2H, Ar-*H*), 7.13 (td, *J* = 7.5, 1.6 Hz, 1H, Ar-*H*), 5.92 (s, 1H, C*H*), 4.21–3.94 (m, 4H, C*H$_2$*). **^{13}C{^{1}H} NMR** (126 MHz, $CDCl_3$) δ 139.6, 139.1 (d, *J* = 3.9 Hz), 136.7, 132.1 (q, *J* = 33.2 Hz), 130.4, 127.9, 127.9, 122.9 (q, *J* = 273.3 Hz), 122.1 (p, *J* = 3.9 Hz), 118.4, 115.8, 105.3, 65.1. **^{19}F NMR** (376 MHz, $CDCl_3$) δ -62.92 (s). **^{125}Te{^{1}H} NMR** (126 MHz, $CDCl_3$) δ 674.1 (s).

Synthesis of compound **d**

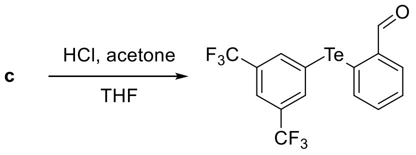

d

An HCl solution (3% w/w in H_2O, 10 mL) was added to a solution of compound **c** (3.70 g, 7.55 mmol) in THF (40 mL) and acetone (20 mL). The mixture was stirred for 30 min and then extracted with Et_2O (3 × 30 mL). The combined organic phases were dried over $MgSO_4$, filtered, and brought to dryness under vacuum to afford a yellow solid. The crude mixture was purified by 100–200 mesh silica gel flash chromatography (Hexanes/CH_2Cl_2 = 7:3 as eluents) to give compound **d** as a yellow solid (2.92 g, 6.55 mmol, 87% yield). **^{1}H NMR** (500 MHz, $CDCl_3$) δ 10.22 (s, 1H, C*H*O), 8.39 (s, 2H, Ar-*H*), 7.96 (s, 1H, Ar-*H*), 7.94 (dd, *J* = 7.6, 1.6 Hz, 1H, Ar-*H*), 7.42 (td, *J* = 7.4, 1.1 Hz, 1H, Ar-*H*), 7.31 (td, *J* = 7.5, 1.6 Hz, 1H, Ar-*H*), 7.04 (d, *J* = 7.9 Hz, 1H, Ar-*H*). **^{13}C{^{1}H} NMR** (126 MHz, $CDCl_3$) δ 193.2, 141.4 (d, *J* = 3.7 Hz), 137.0, 136.0, 134.6, 133.2, 132.6 (q, *J* = 33.3 Hz), 126.7, 125.9, 123.1 (p, *J* = 3.7 Hz), 123.1 (q, *J* = 273.2 Hz), 119.2. **^{19}F NMR** (376 MHz, $CDCl_3$) δ -62.78 (s). **^{125}Te{^{1}H} NMR** (126 MHz, $CDCl_3$) δ 814.4 (s).

Synthesis of compound **e**

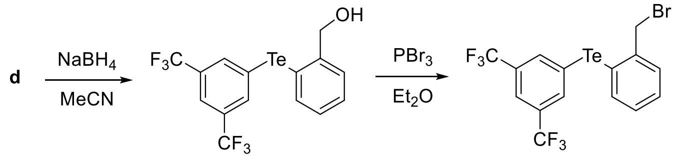

d^{OH} **e**

$NaBH_4$ (744 mg, 19.65 mmol) was added in portions to a solution of compound **d** (2.92 g, 6.55 mmol) in acetonitrile (20 mL). The resulting mixture was stirred for 3 h and then slowly quenched with an HCl solution (3% w/w in H_2O, 20 mL) at 0 °C. The aqueous layer was extracted with CH_2Cl_2 (3 × 30 mL). The combined organic phases were dried over $MgSO_4$, filtered, and brought to dryness under vacuum to afford a dark yellow oil. The

crude mixture was purified by 100–200 mesh silica gel flash chromatography (CH_2Cl_2/MeOH = 9:1 as eluents) to give compound $\mathbf{d}^{OH}$ as a plain yellow solid (2.80 g, 6.25 mmol, 96% yield). **^{1}H NMR** (500 MHz, $CDCl_3$) δ 8.11 (s, 2H, Ar-*H*), 7.79 (s, 1H, Ar-*H*), 7.51 (dd, *J* = 7.7, 1.3 Hz, 1H, Ar-*H*), 7.41 (dd, *J* = 7.6, 1.6 Hz, 1H, Ar-*H*), 7.32 (td, *J* = 7.5, 1.3 Hz, 1H, Ar-*H*), 7.13 (td, *J* = 7.5, 1.6 Hz, 1H, Ar-*H*), 4.79 (d, *J* = 5.7 Hz, 2H, C*H₂*), 1.93 (t, *J* = 5.8 Hz, 1H, O*H*). **^{13}C{^{1}H} NMR** (126 MHz, $CDCl_3$) δ 143.9, 138.3, 138.1 (d, *J* = 3.9 Hz), 132.3 (q, *J* = 33.2 Hz), 129.5, 128.9, 128.7, 123.0 (q, *J* = 273.2 Hz), 122.0 (p, *J* = 3.8 Hz), 118.3, 117.9, 68.7. **^{19}F NMR** (376 MHz, $CDCl_3$) δ -62.93 (s). **^{125}Te{^{1}H} NMR** (126 MHz, $CDCl_3$) δ 652.5 (s).

Under a nitrogen atmosphere, PBr_3 (0.65 mL, 1.86 g, 6.88 mmol) was slowly added to a solution of compound $\mathbf{d}^{OH}$ (2.80 g, 6.25 mmol) in anhydrous Et_2O (30 mL) at 0 °C. The resulting mixture was allowed to stir overnight at room temperature and then slowly quenched with a saturated aqueous solution of Na_2CO_3 (30 mL) at 0 °C. The aqueous layer was extracted with Et_2O (3 × 30 mL). The combined organic phases were dried over $MgSO_4$, filtered, and brought to dryness under vacuum to afford a yellow residue. The crude product was purified by 100–200 mesh silica gel flash chromatography (Hexanes/CH_2Cl_2 = 8:2 as eluent) to give compound **e** as a yellow solid (2.27 g, 4.44 mmol, 71% yield). **^{1}H NMR** (500 MHz, $CDCl_3$) δ 8.03 (s, 2H, Ar-*H*), 7.78–7.74 (m, 2H, Ar-*H*), 7.56 (dd, *J* = 7.7, 1.5 Hz, 1H, Ar-*H*), 7.39 (td, *J* = 7.6, 1.4 Hz, 1H, Ar-*H*), 7.16 (td, *J* = 7.5, 1.5 Hz, 1H, Ar-*H*), 4.76 (s, 1H, 2H, C*H₂*).**^{13}C{^{1}H} NMR** (126 MHz, $CDCl_3$) δ 142.7, 141.2, 137.0 (d, *J* = 3.7 Hz), 132.4 (q, *J* = 33.3 Hz), 130.28, 130.23, 130.18, 123.0 (q, *J* = 273.2 Hz), 121.9(p, *J* = 3.8 Hz), 118.7, 117.8, 39.0. **^{19}F NMR** (376 MHz, $CDCl_3$) δ -62.99 (s). **^{125}Te{^{1}H} NMR** (126 MHz, $CDCl_3$) δ 639.4 (s).

Synthesis of compound **1**

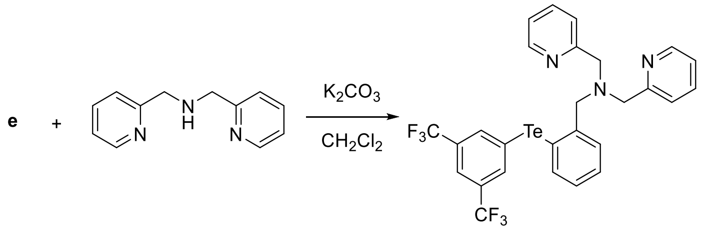

1

K_2CO_3 (2.35 g, 17.03 mmol) was added to a solution of compound **e** (2.89 g, 5.68 mmol) and dipicolylamine (1.13 g, 5.68 mmol) in CH_2Cl_2 (20 mL). The resulting mixture was stirred overnight, filtered through Celite, and brought to dryness under vacuum to afford a dark yellow residue. The crude product was purified by 100–200 mesh silica gel flash chromatography (CH_2Cl_2/MeOH = 98:2 as eluents) to give compound **1** as a beige solid (2.18 g, 3.50 mmol, 62% yield). **^{1}H NMR** (500 MHz, $CDCl_3$) δ 8.59 (dd, *J* = 4.89, 1.80 Hz, 2H, Ar-*H*), 8.25 (s, 2H, Ar-*H*), 7.81 (s, 1H, Ar-*H*), 7.67 (td, *J* = 7.64, 1.88 Hz, 2H, Ar-*H*), 7.45 (dd, *J* = 7.81, 1.22 Hz, 2H, Ar-*H*), 7.24 (dd, *J* = 7.57, 1.54 Hz, 1H, Ar-*H*), 7.22–7.13 (m, 4H, Ar-*H*), 6.99 (td, *J* = 7.56, 1.58 Hz, 1H, Ar-*H*), 3.94 (s, 2H, C*H₂*), 3.81 (s, 4H, C*H₂*).

$^{13}C\{^1H\}$ NMR (126 MHz, $CDCl_3$) δ 157.5, 149.2, 141.8, 139.5, 139.4, 136.3, 135.3, 131.9 (q, J = 33.0 Hz), 130.4, 128.7, 127.2, 125.0, 123.2 (q, $^1J_{C-F}$ = 273.2 Hz), 122.4, 121.9 – 121.7 (m), 121.6, 62.5, 58.0. **^{19}F NMR** (376 MHz, $CDCl_3$) δ -62.79 (s). **$^{125}Te\{^1H\}$ NMR** (126 MHz, CD_2Cl_2) δ 683.7 (s). **Elemental Analysis** $C_{27}H_{21}F_6N_3Te$ calculated: C: 51.55; H: 3.36. Found: C: 51.28; H: 3.27.

Synthesis of compound **2**

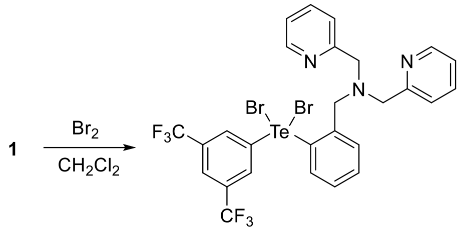

2

A solution of bromine (0.1 mL, 3.2 M in CH_2Cl_2) was added dropwise to a solution of compound **1** (200 mg, 0.32 mmol) in CH_2Cl_2 (5 mL). The mixture was stirred for 5 min and then brought to dryness under vacuum. The crude product was washed with hexanes (3 × 10 mL) to afford compound **2** as a yellow powder (232 mg, 0.29 mmol, 93% yield). **^1H NMR** (500 MHz, $CDCl_3$) δ 8.84 (s, 2H, Ar-*H*), 8.76 z(dd, J = 4.97, 1.86 Hz, 2H, Ar-*H*), 8.00 (s, 1H, Ar-*H*), 7.63 (td, J = 7.62, 1.89 Hz, 2H, Ar-*H*), 7.59 (d, J = 7.94 Hz, 1H, Ar-*H*), 7.54 (t, J = 7.36 Hz, 1H, Ar-*H*), 7.45–7.35 (m, 2H, Ar-*H*), 7.26–7.23 (m, 2H, Ar-*H*), 7.09 (d, J = 7.71 Hz, 2H, Ar-*H*), 4.61 (s, 2H, C*H₂*), 4.18ii (s, 4H, C*H₂*). **$^{13}C\{^1H\}$ NMR** (126 MHz, $CDCl_3$) δ 155.9, 149.6, 140.4, 140.2, 137.3, 136.5, 135.7, 134.2, 132.0 (q, J = 33.5 Hz), 131.8, 130.4, 130.1, 124.9, 124.3, 123.1 (q, $^1J_{C-F}$ = 273.0 Hz), 122.7, 57.1, 55.9. **^{19}F NMR** (376 MHz, $CDCl_3$) δ -62.58 (s). **$^{125}Te\{^1H\}$ NMR** (126 MHz, CD_2Cl_2) δ 962.7 (s). **Elemental Analysis** $C_{27}H_{21}Br_2F_6N_3Te$ calculated: C: 41.11; H: 2.68. Found: C: 40.87; H: 2.57.

Synthesis of compound **1**-$ZnCl_2$

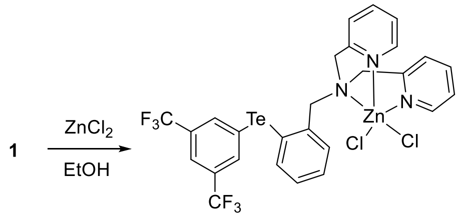

1-$ZnCl_2$

$ZnCl_2$ (11 mg, 0.08 mmol) was added to a solution of compound **1** (50 mg, 0.08 mmol) in ethanol (3 mL). The resulting mixture was stirred for 5 min and then brought to dryness under vacuum to afford compound **1**-$ZnCl_2$ as a white powder (56 mg, 0.07 mmol, 92% yield). **^1H NMR** (500 MHz, $CDCl_3$) δ 9.24 (dd, J = 5.35, 1.69 Hz, 2H, Ar-*H*), 7.83 (td, J = 7.63, 1.71 Hz, 2H, Ar-*H*), 7.75 (s, 2H, Ar-*H*), 7.71 (s, 1H, Ar-*H*), 7.59 (dd, J = 7.67, 1.33 Hz, 1H, Ar-*H*), 7.49 (dd, J = 7.74, 1.51 Hz, 1H, Ar-*H*), 7.48 – 7.43 (m, 2H, Ar-*H*), 7.25 –

7.20 (m, 3H, Ar-*H*), 7.02 (td, $J = 7.57$, 1.51 Hz, 1H, Ar-*H*), 4.28 (s, 4H, C*H*$_2$), 4.13 (s, 2H, C*H*$_2$). **^{13}C{^{1}H} NMR** (126 MHz, $CDCl_3$) δ 154.2, 150.0, 141.0, 140.1, 138.4, 136.2, 132.6, 132.5 (q, $J = 33.3$ Hz), 130.3, 130.1, 124.7, 123.8, 122.8 (q, $^1J_{C\text{-}F} = 273.2$ Hz), 122.0, 119.7, 118.3, 61.9, 57.9. **^{19}F NMR** (376 MHz, $CDCl_3$) δ -62.98 (s). **^{125}Te{^{1}H} NMR** (126 MHz, CD_2Cl_2) δ 652.6 (s).

Synthesis of compound **2**-$ZnBr_2$

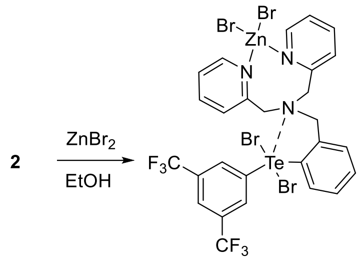

2-$ZnBr_2$

$ZnBr_2$ (14 mg, 0.06 mmol) was added to a solution of compound **2** (50 mg, 0.06 mmol) in ethanol (3 mL). The resulting mixture was stirred for 5 min and then brought to dryness under vacuum to afford compound **2**-$ZnBr_2$ as a white powder (55 mg, 0.05 mmol, 86% yield). **^{1}H NMR** (500 MHz, $CDCl_3$) δ 9.34 (dd, $J = 5.49$, 1.72 Hz, 2H, Ar-*H*), 8.76 (s, 2H, Ar-*H*), 8.09 – 8.00 (m, 3H, Ar-*H*), 7.69 (ddd, $J = 7.35$, 5.50, 1.31 Hz, 2H, Ar-*H*), 7.63 (d, $J = 7.78$ Hz, 2H, Ar-*H*), 7.54 (td, $J = 7.42$, 1.25 Hz, 1H, Ar-*H*), 7.49 (dd, $J = 8.11$, 1.24 Hz, 1H, Ar-*H*), 7.45 (dd, $J = 7.73$, 1.52 Hz, 1H, Ar-*H*), 4.34 (s, 4H, C*H*$_2$), 4.33 (s, 2H, C*H*$_2$). **^{13}C{^{1}H} NMR** (126 MHz, $CDCl_3$) δ 153.5, 151.0, 141.3, 137.6, 136.93, 136.90, 135.7, 135.1, 134.3, 132.8 (q, $J = 34.2$ Hz), 132.5, 131.5, 131.2, 126.0, 122.7 (q, $^1J_{C\text{-}F} = 273.7$ Hz) 119.4, 58.4, 55.3. **^{19}F NMR** (376 MHz, $CDCl_3$) δ -62.71 (s). The ^{125}Te{^{1}H} NMR resonance could not be detected, reflecting the low solubility of compound **2-**$ZnBr_2$ in CD_2Cl_2.

1.3 NMR Spectra

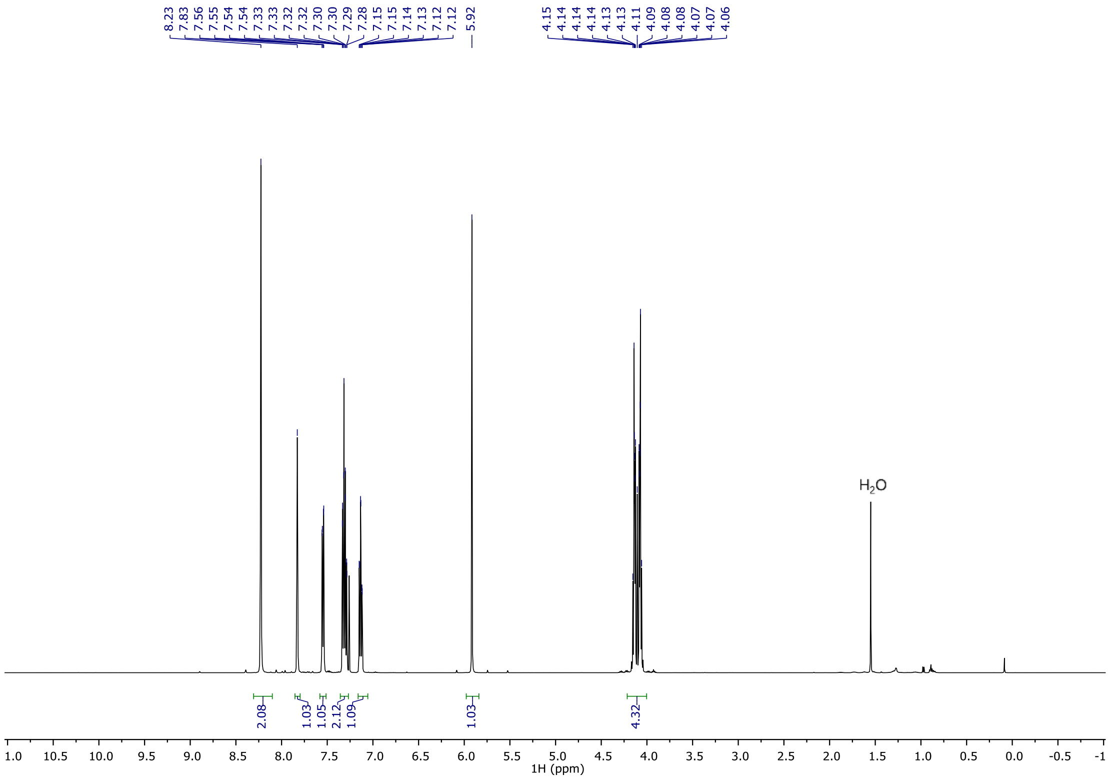

Figure S1. ^{1}H NMR spectrum of compound **c** in $CDCl_3$.

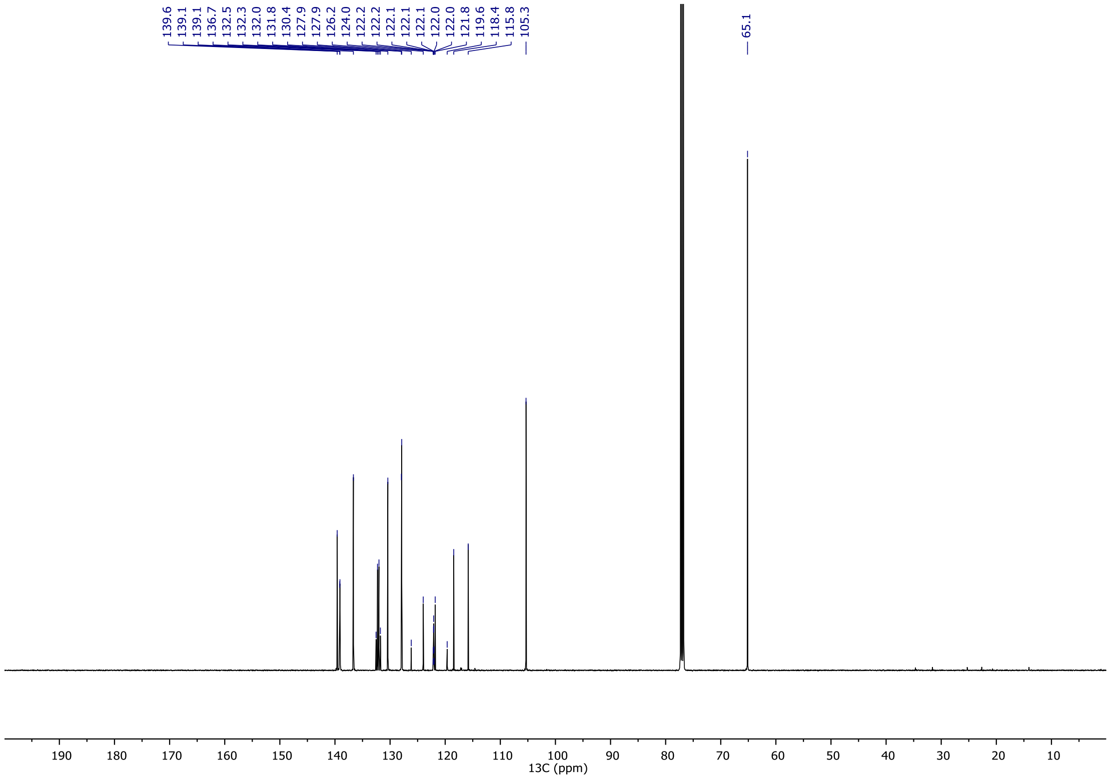

Figure S2. ^{13}C{^{1}H} NMR spectrum of compound **c** in $CDCl_3$.

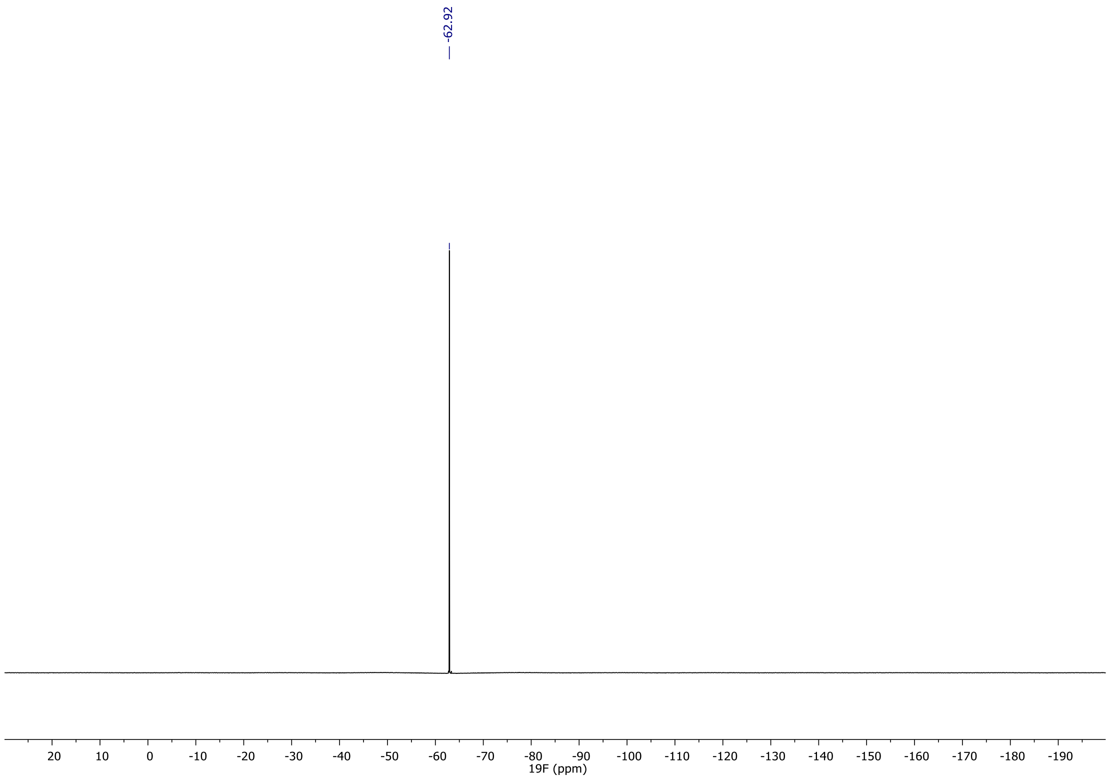

Figure S3. ^{19}F NMR spectrum of compound **c** in $CDCl_3$.

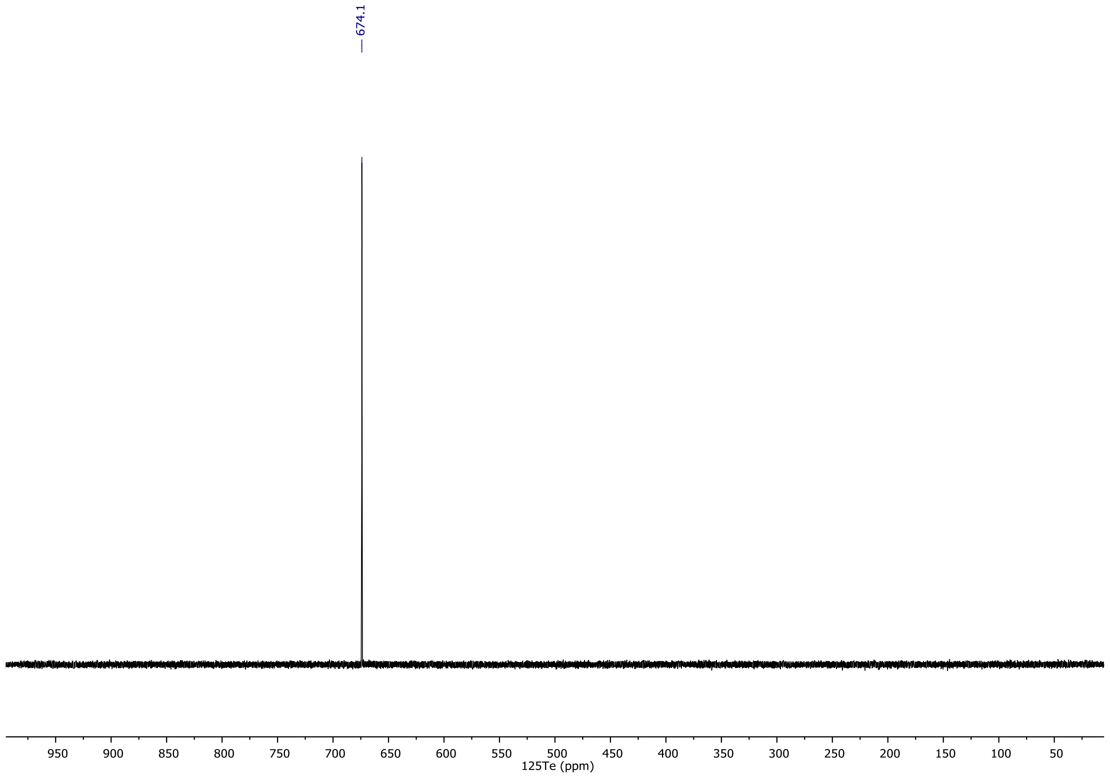

Figure S4. ^{125}Te{^{1}H} NMR spectrum of compound **c** in $CDCl_3$.

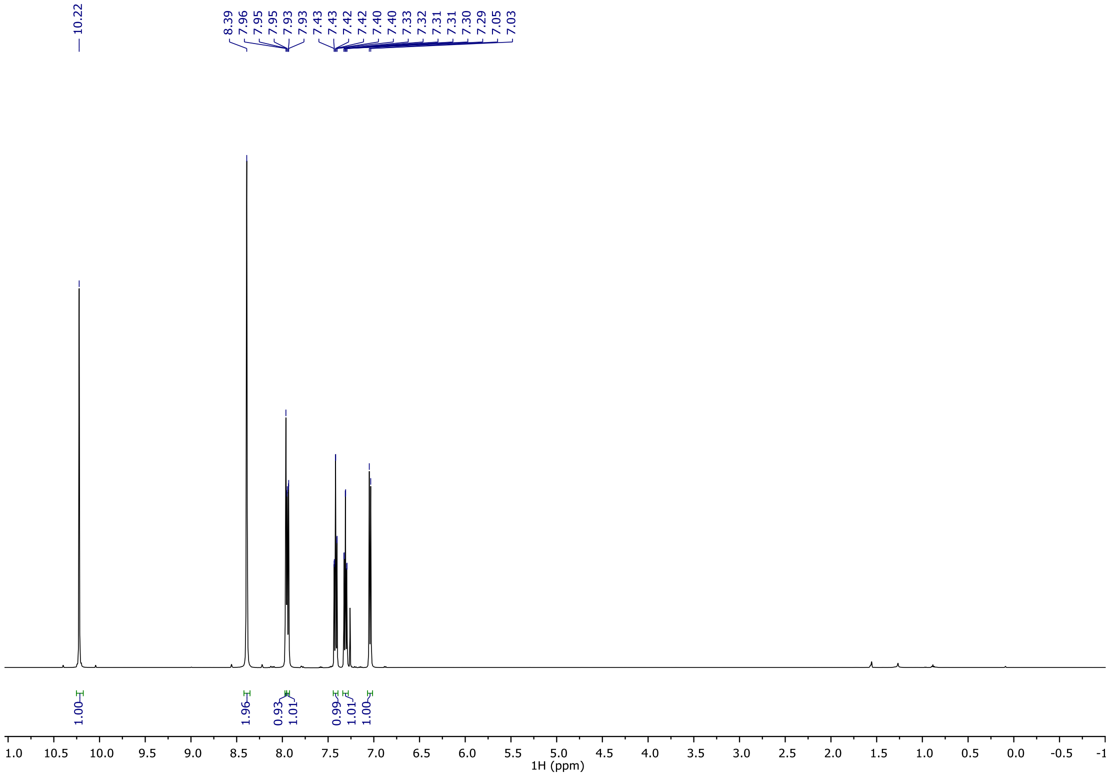

Figure S5. ^{1}H NMR spectrum of compound **d** in $CDCl_3$.

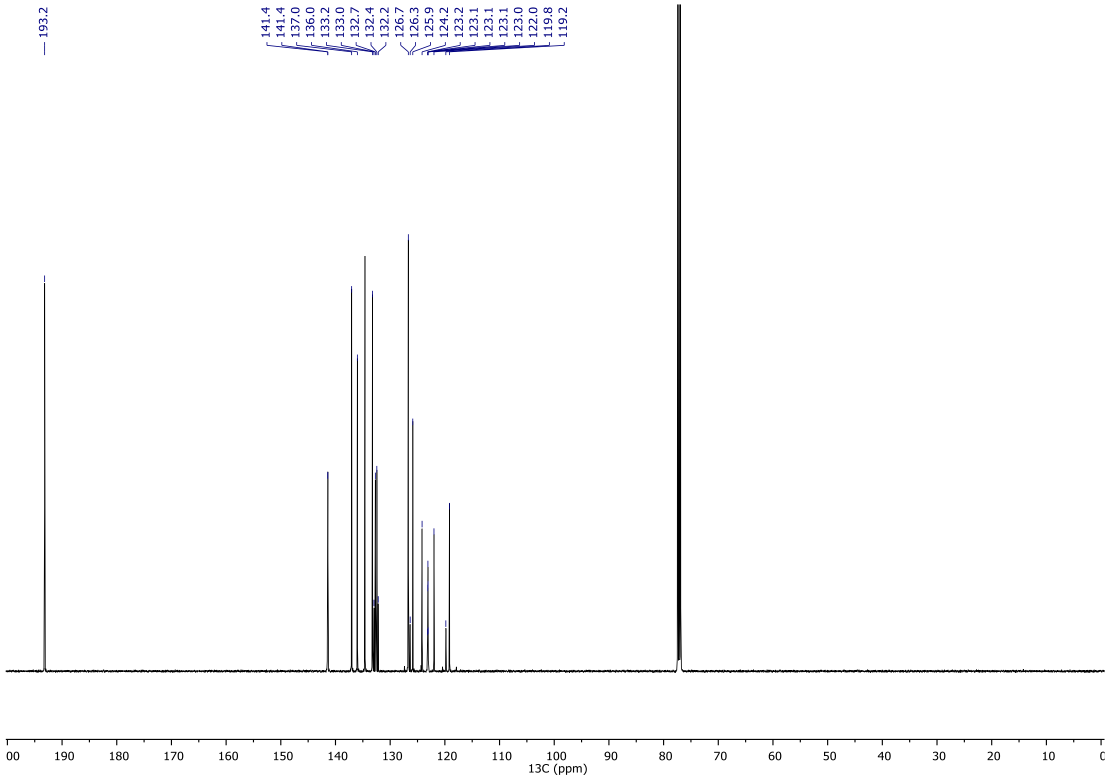

Figure S6. $^{13}C\{^{1}H\}$ NMR spectrum of compound **d** in $CDCl_3$.

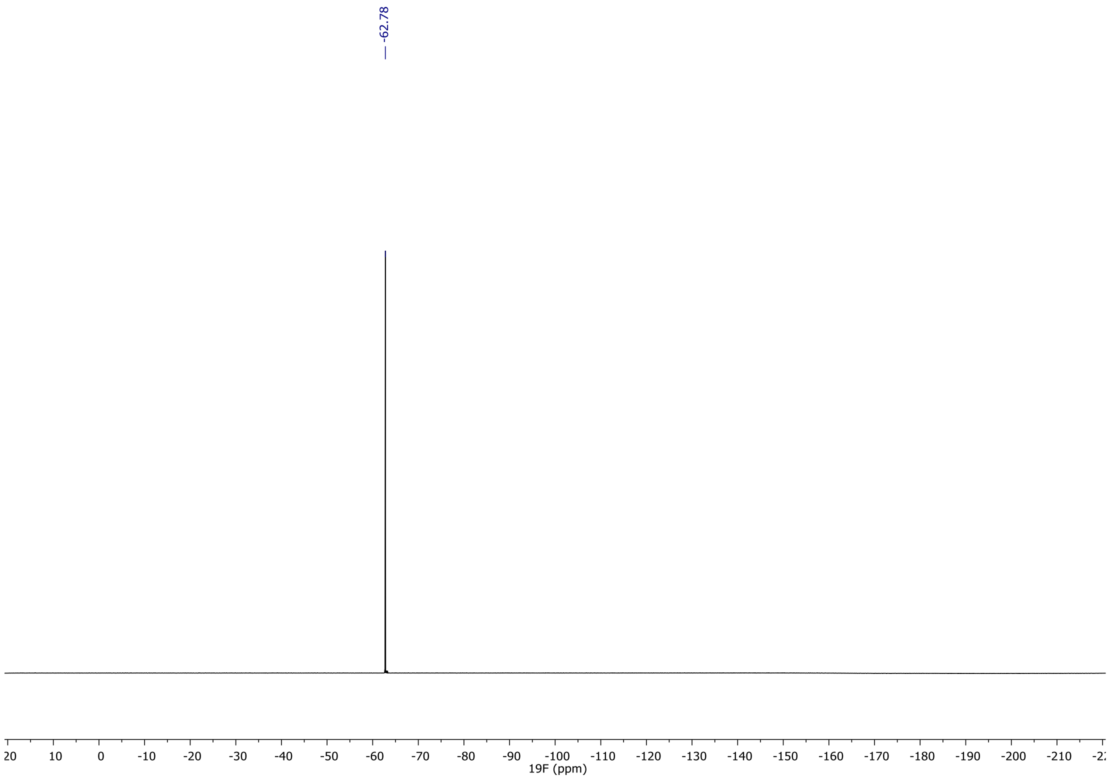

Figure S7. ^{19}F NMR spectrum of compound **d** in $CDCl_3$.

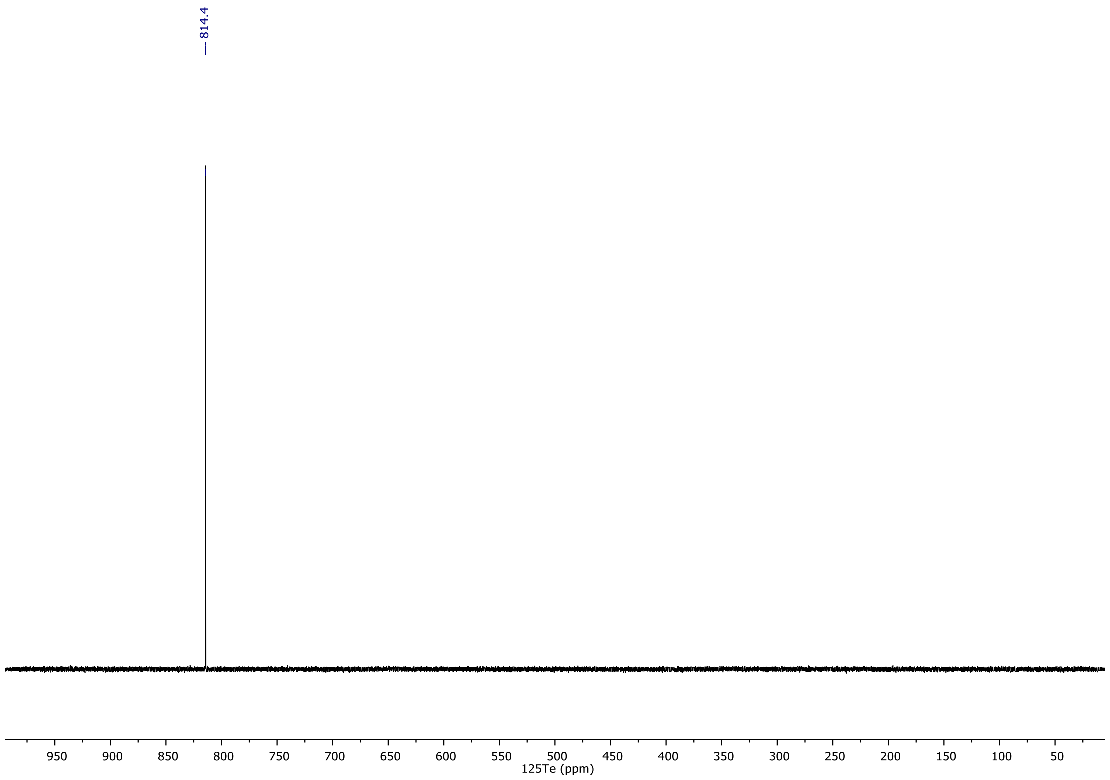

Figure S8. [125]Te{^{1}H} NMR spectrum of compound **d** in $CDCl_3$.

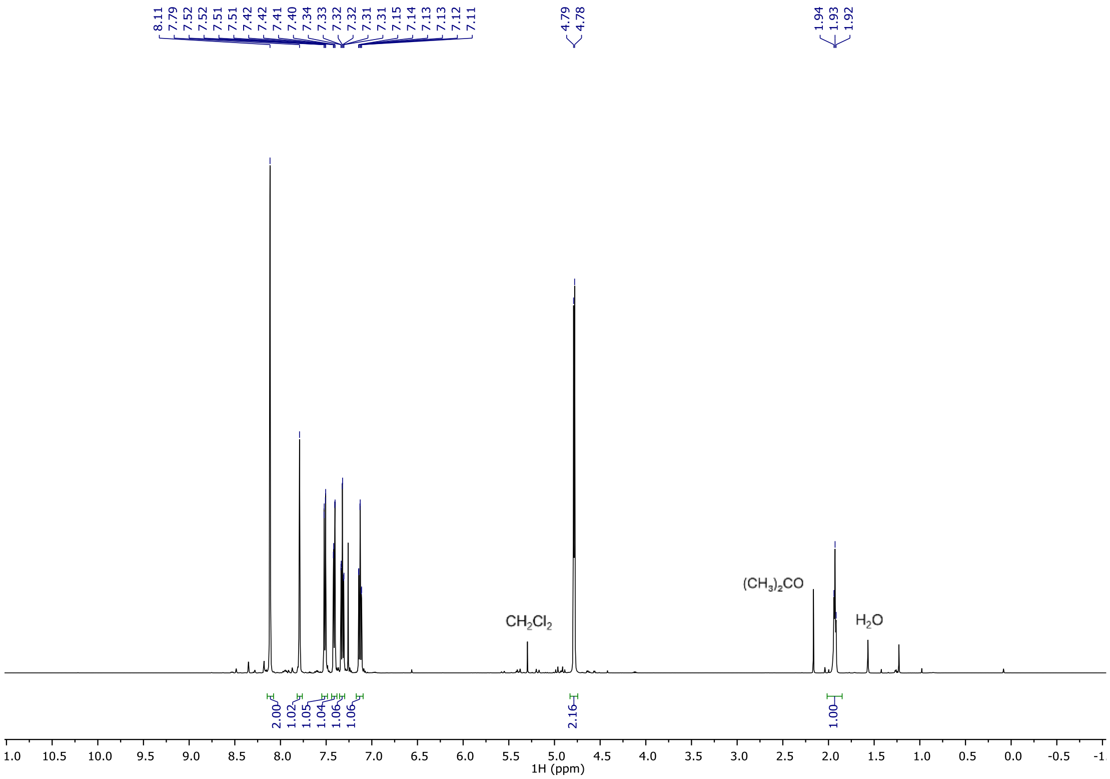

Figure S9. ^{1}H NMR spectrum of compound $\mathbf{d}^{OH}$ in $CDCl_3$.

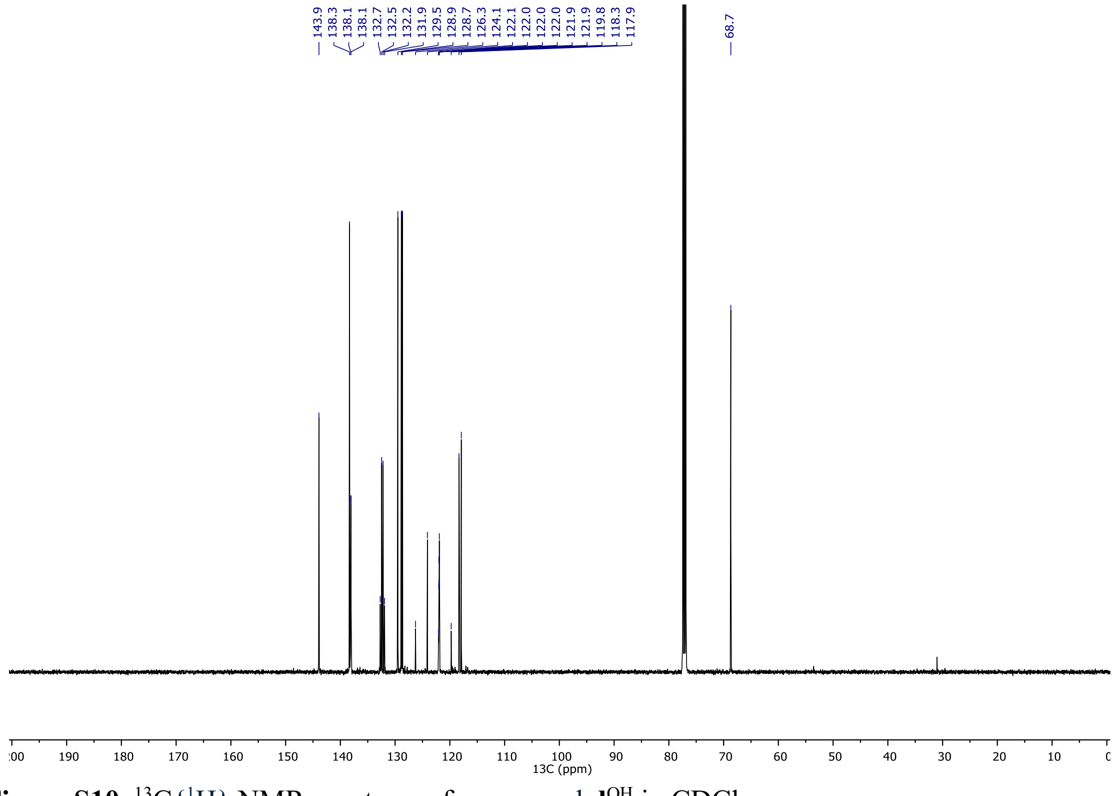

Figure S10. $^{13}C\{^1H\}$ NMR spectrum of compound $\mathbf{d}^{OH}$ in $CDCl_3$.

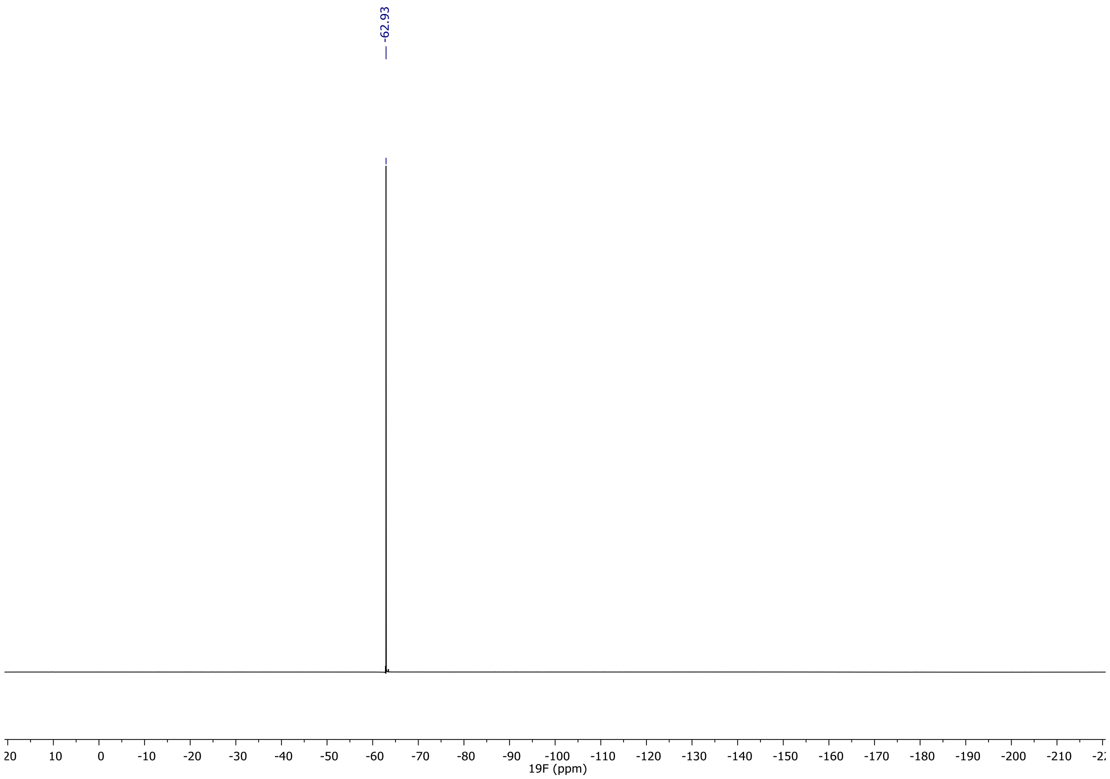

Figure S11. ^{19}F NMR spectrum of compound **d**^{OH} in $CDCl_3$.

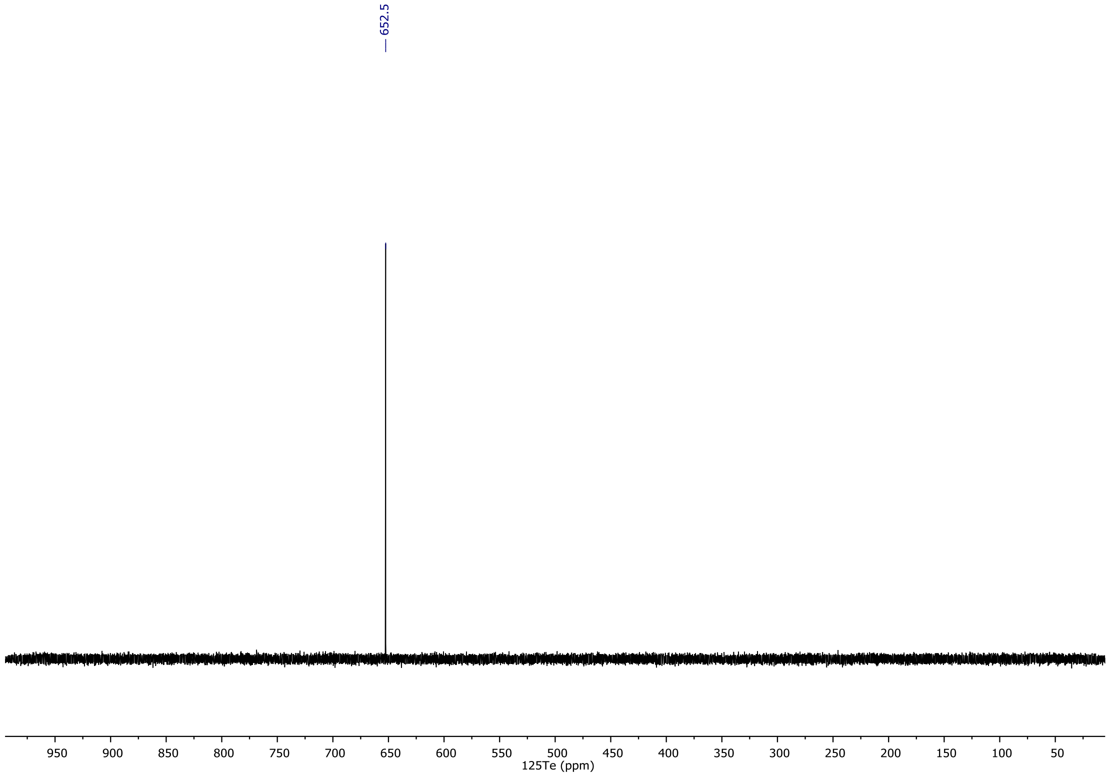

Figure S12. ^{125}Te{^{1}H} NMR spectrum of compound **d**^{OH} in $CDCl_3$.

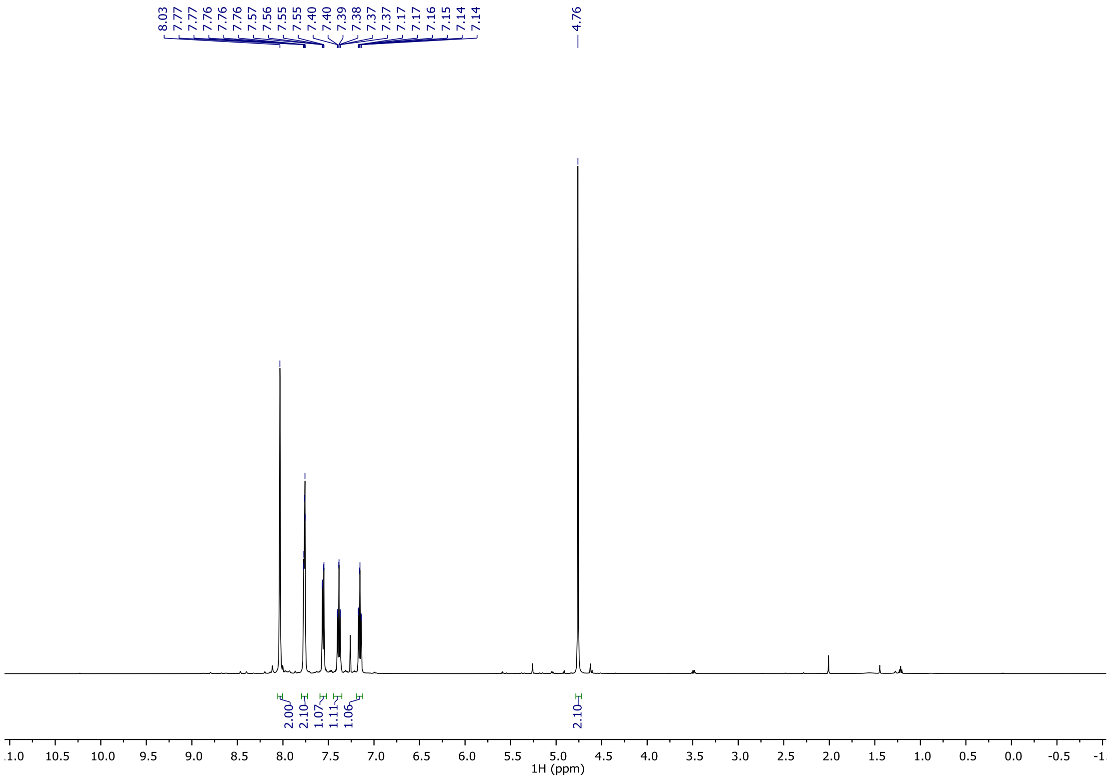

Figure S13. ^{1}H NMR spectrum of compound **e** in $CDCl_3$.

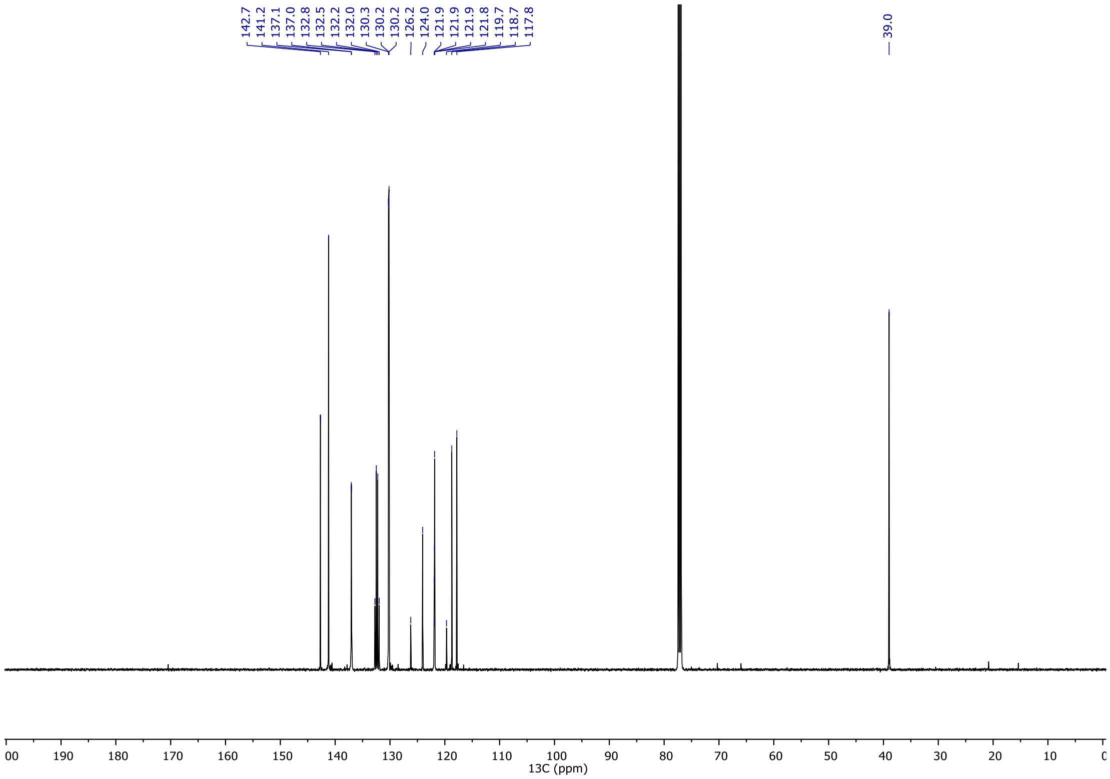

Figure S14. $^{13}C\{^{1}H\}$ NMR spectrum of compound **e** in $CDCl_3$.

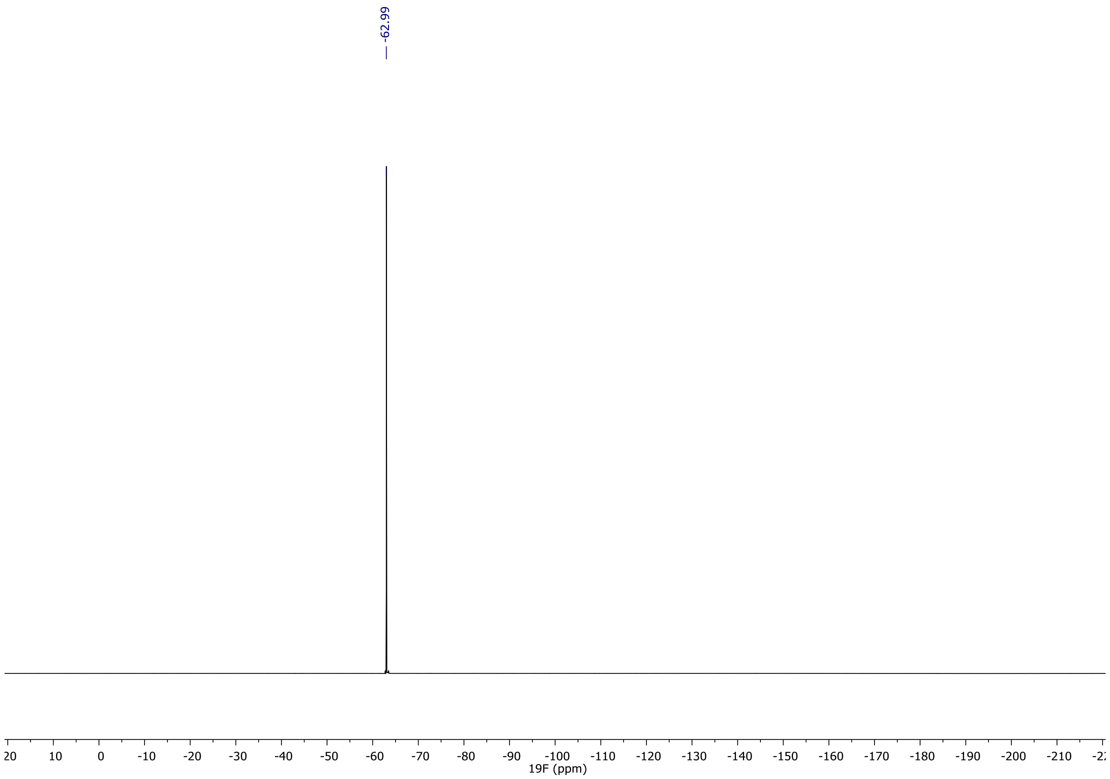

Figure S15. ^{19}F NMR spectrum of compound **e** in $CDCl_3$.

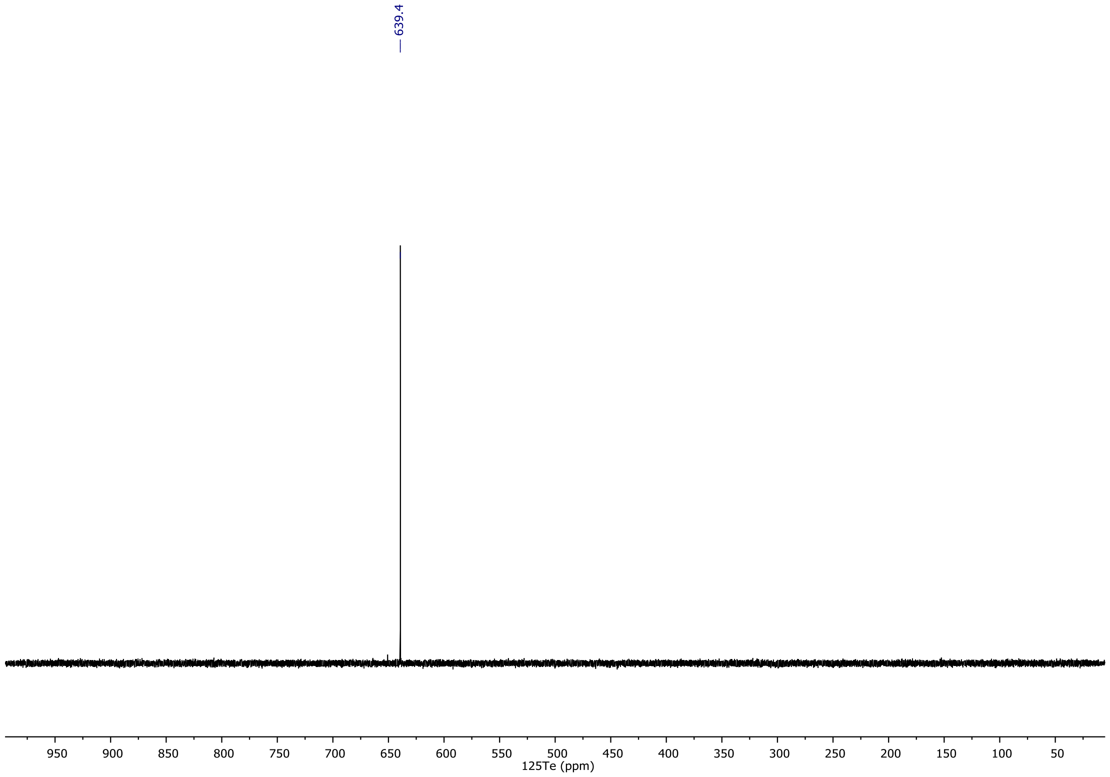

Figure S16. ^{125}Te{^{1}H} NMR spectrum of compound **e** in $CDCl_3$.

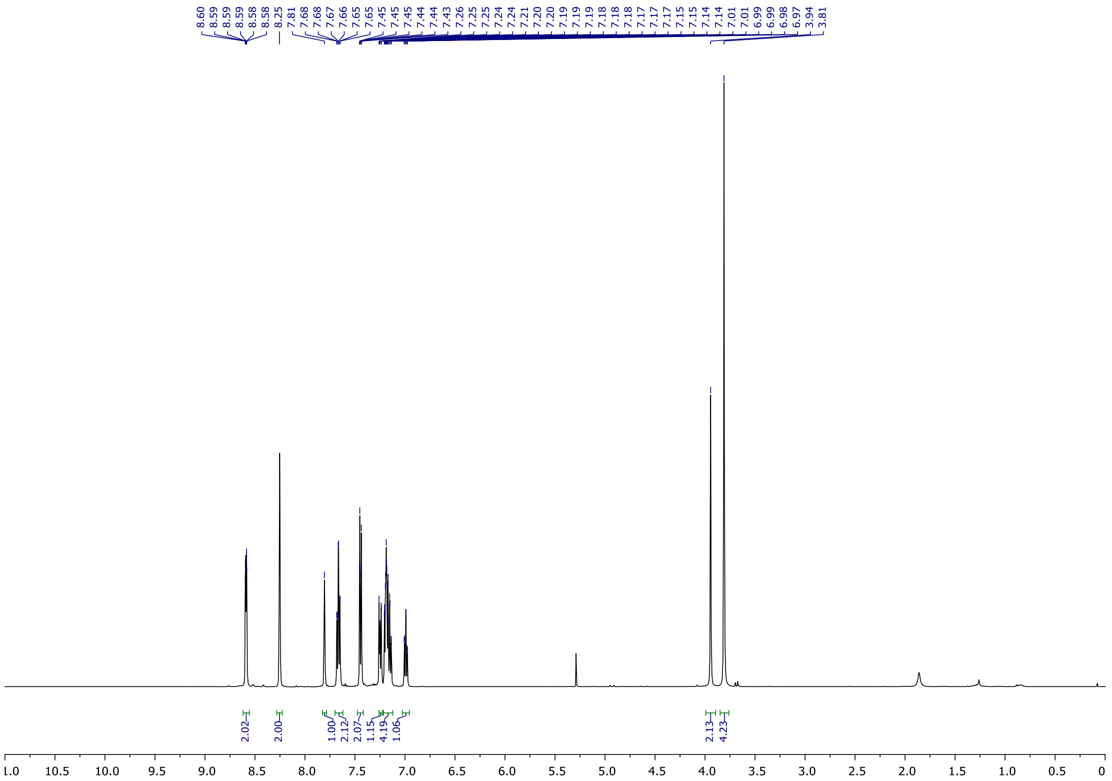

Figure S17. ^{1}H NMR spectrum of compound **1** in $CDCl_3$.

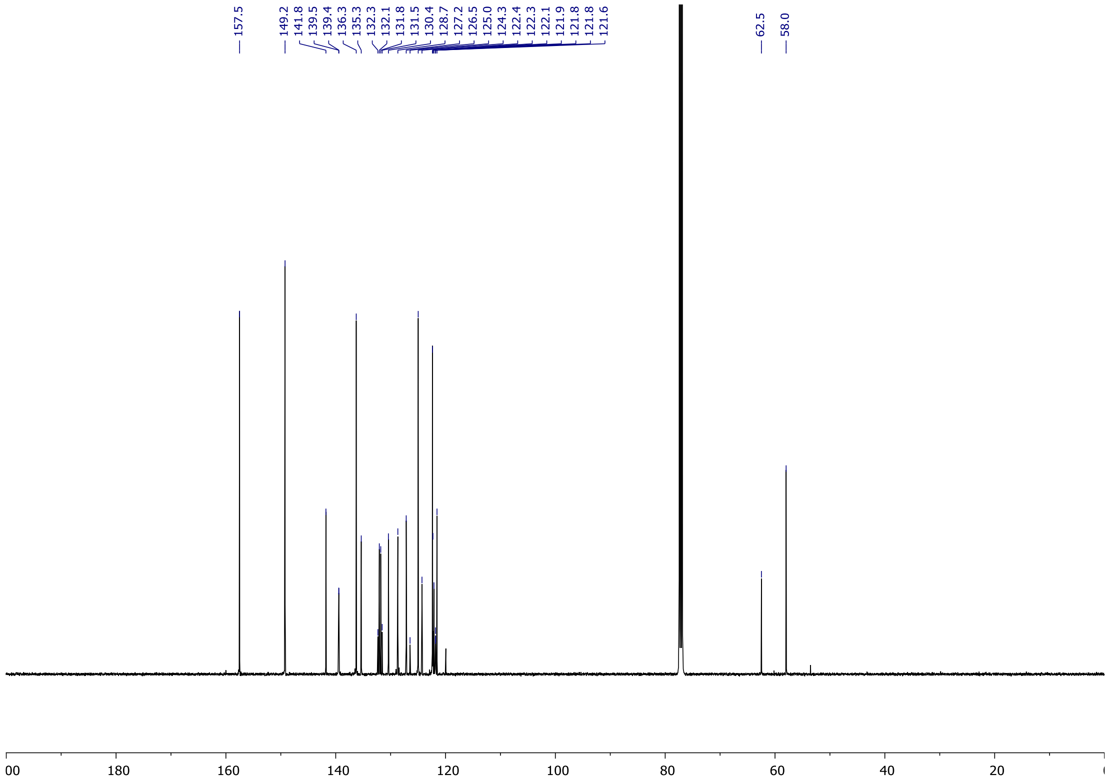

Figure S18. $^{13}C\{^{1}H\}$ NMR spectrum of compound **1** in $CDCl_3$.

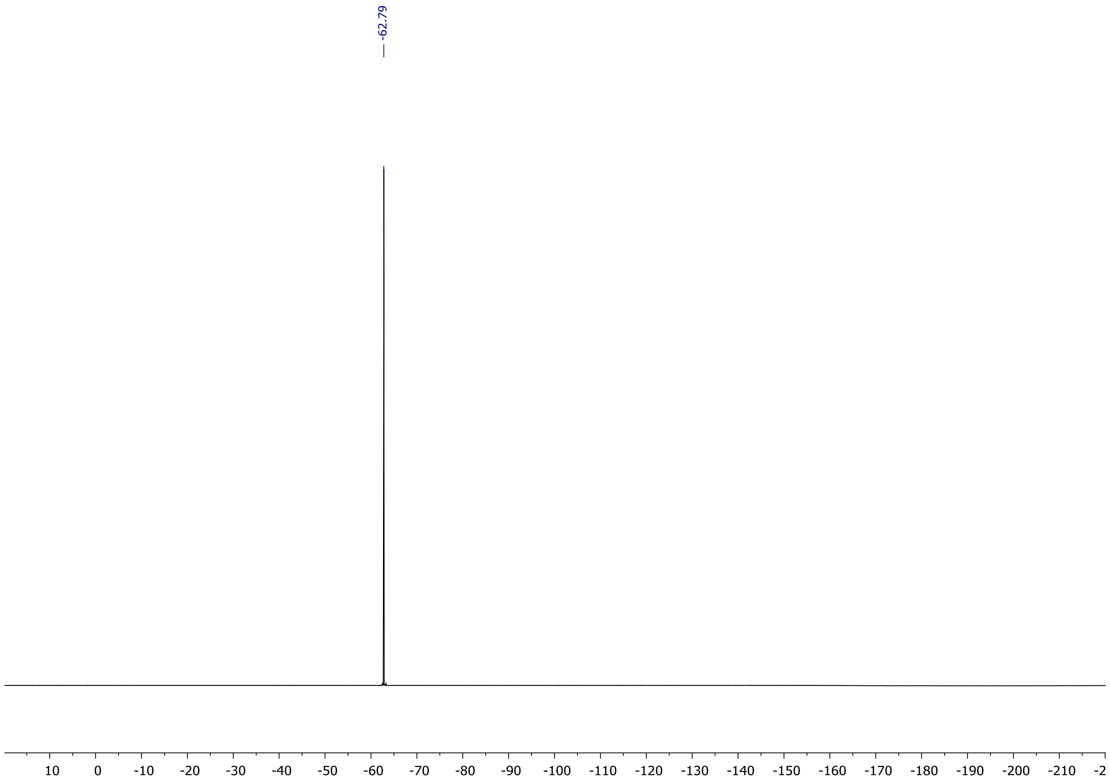

Figure S19. ^{19}F NMR spectrum of compound **1** in $CDCl_3$.

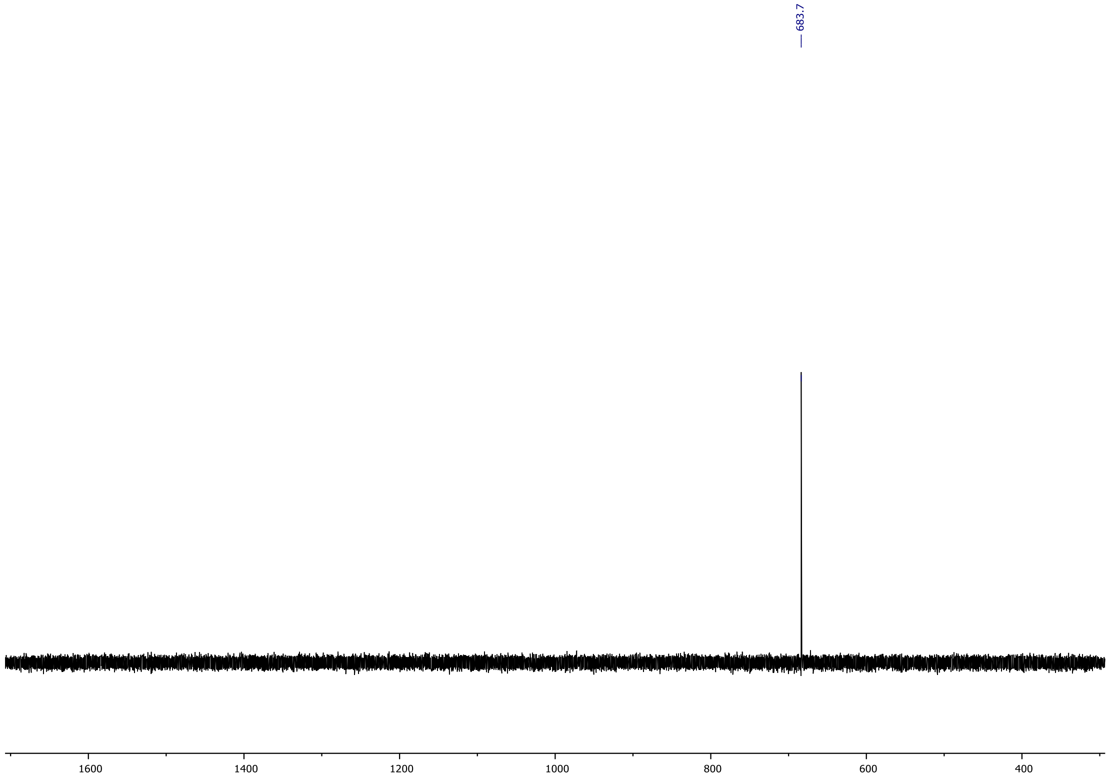

Figure S20. ^{125}Te{^{1}H} NMR spectrum of compound **1** in CD_2Cl_2.

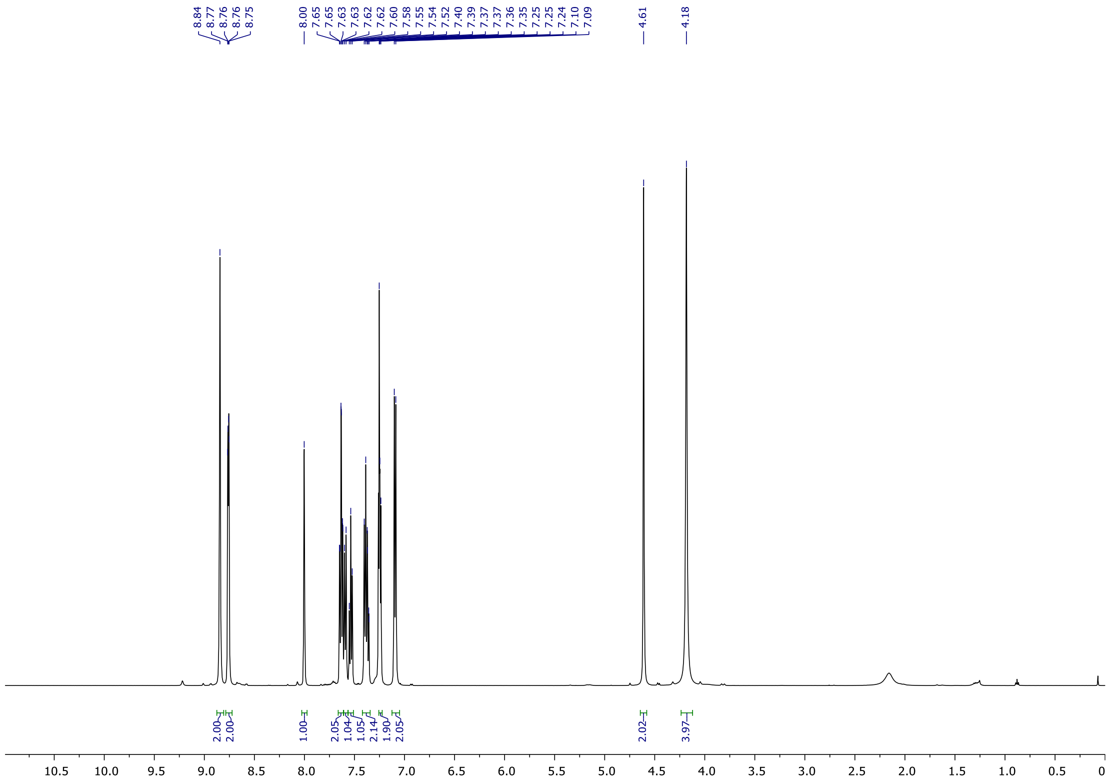

Figure S21. ^{1}H NMR spectrum of compound **2** in $CDCl_3$.

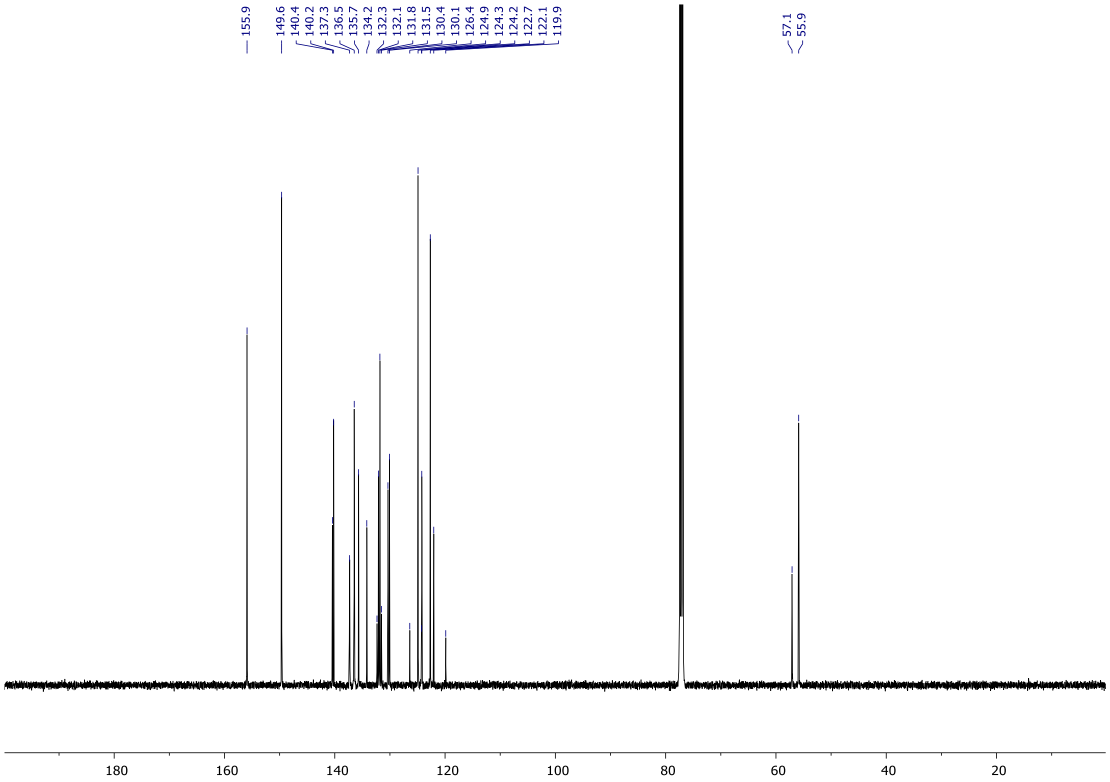

Figure S22. $^{13}C\{^{1}H\}$ NMR spectrum of compound **2** in $CDCl_3$.

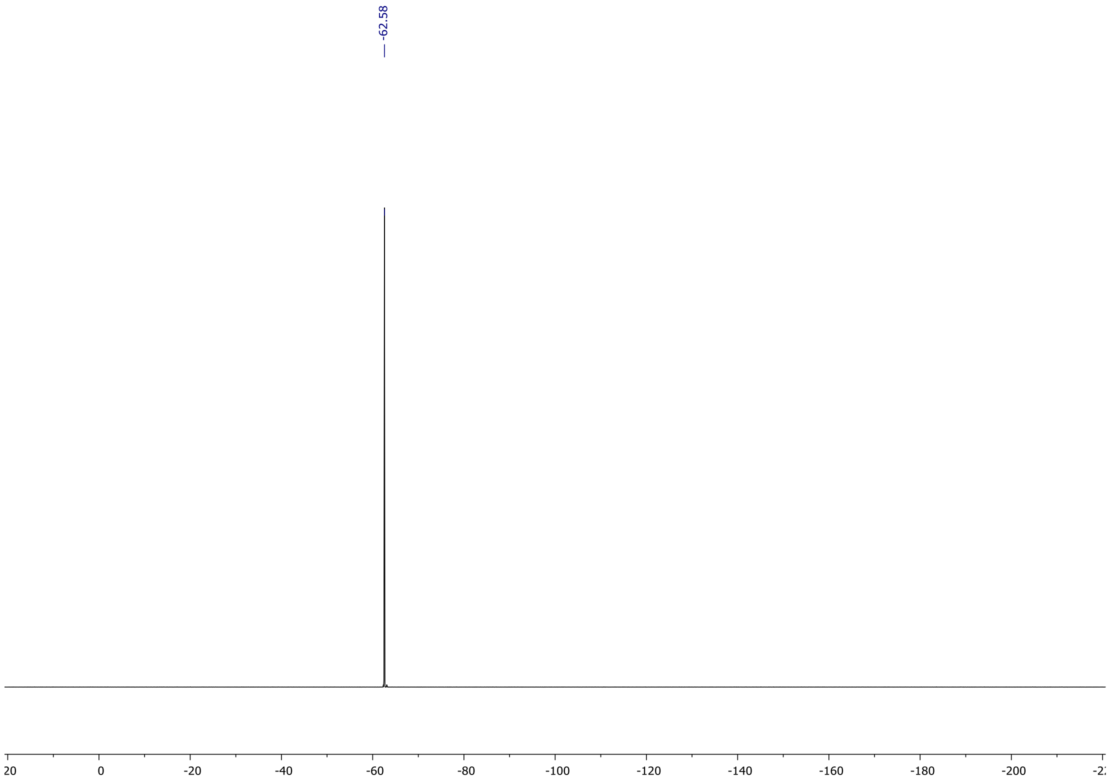

Figure S23. ^{19}F NMR spectrum of compound **2** in $CDCl_3$.

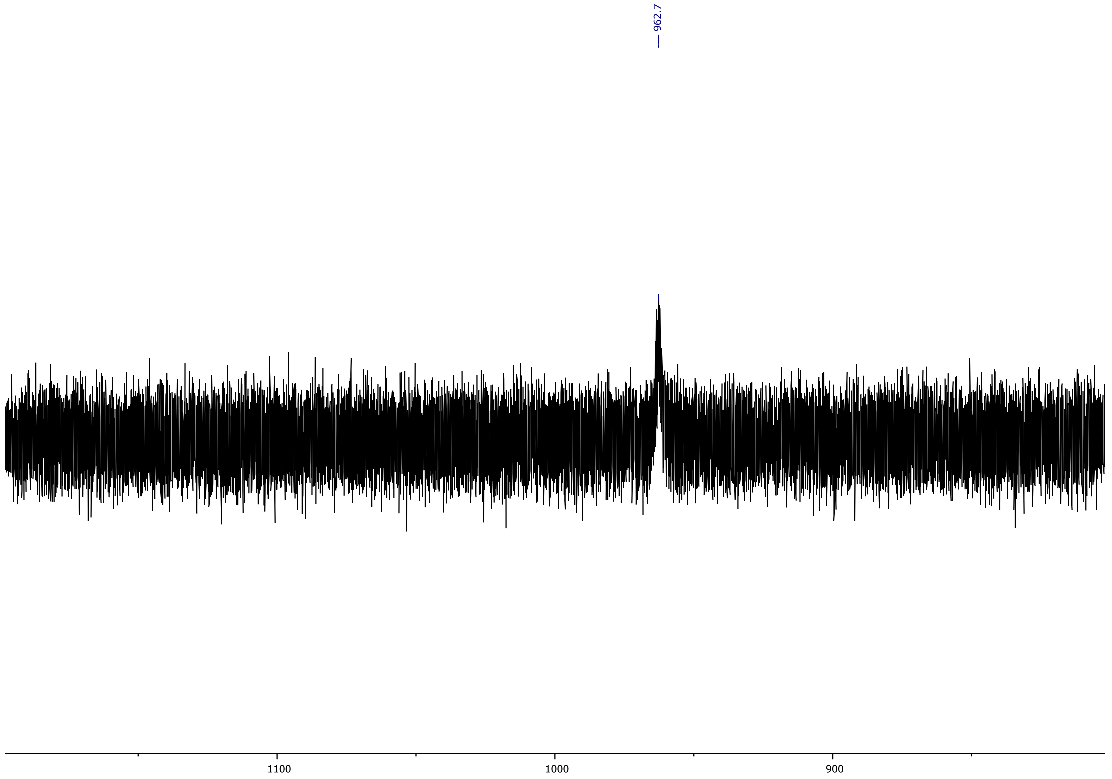

Figure S24. ^{125}Te{^{1}H} NMR spectrum of compound **2** in CD_2Cl_2.

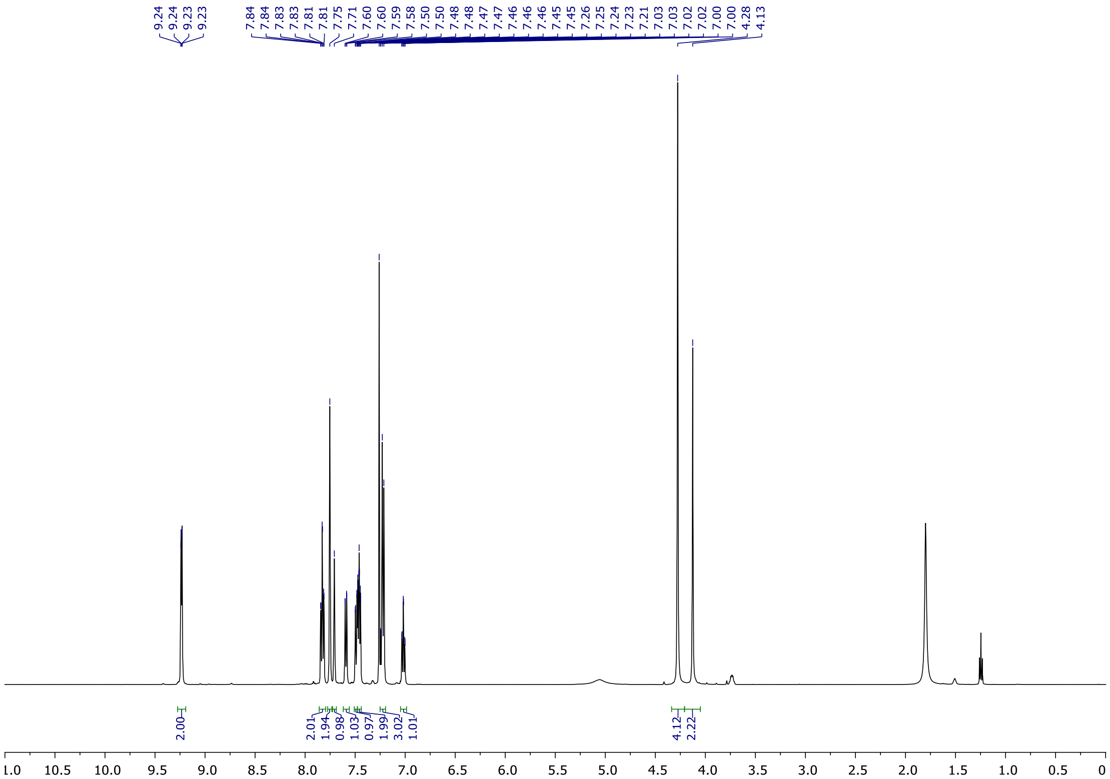

Figure S25. ^{1}H NMR spectrum of compound **1**-$ZnCl_2$ in $CDCl_3$.

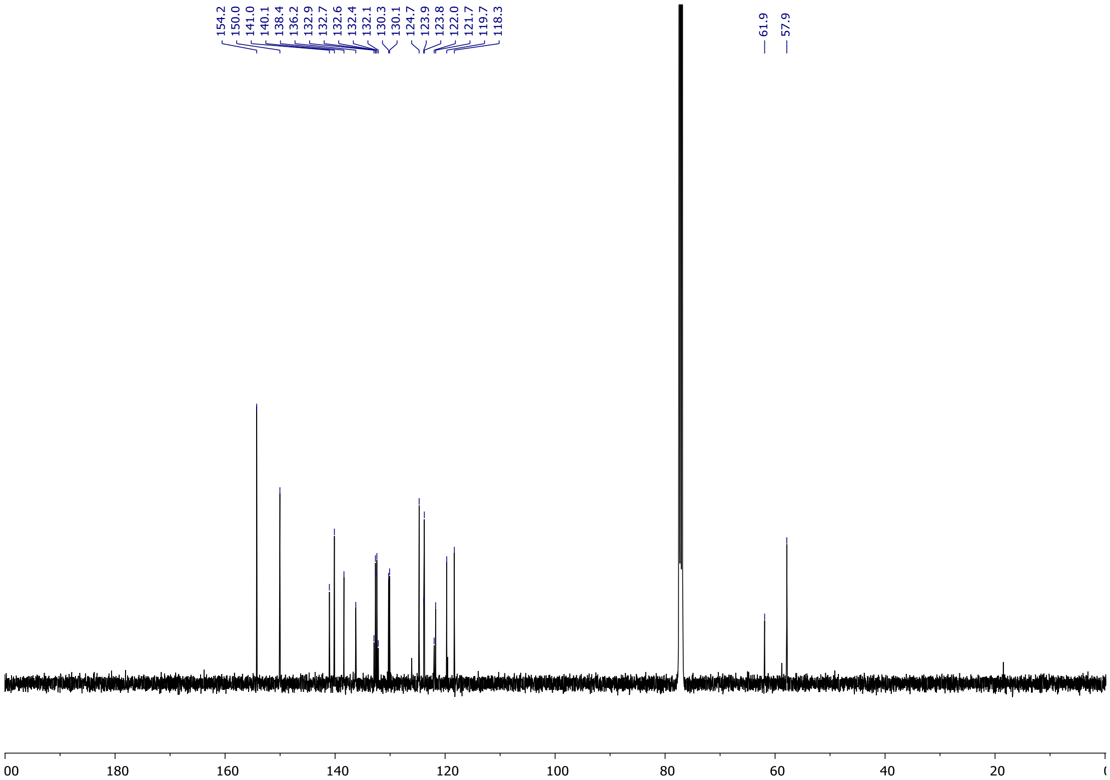

Figure S26. $^{13}C\{^{1}H\}$ NMR spectrum of compound **1**-$ZnCl_2$ in $CDCl_3$.

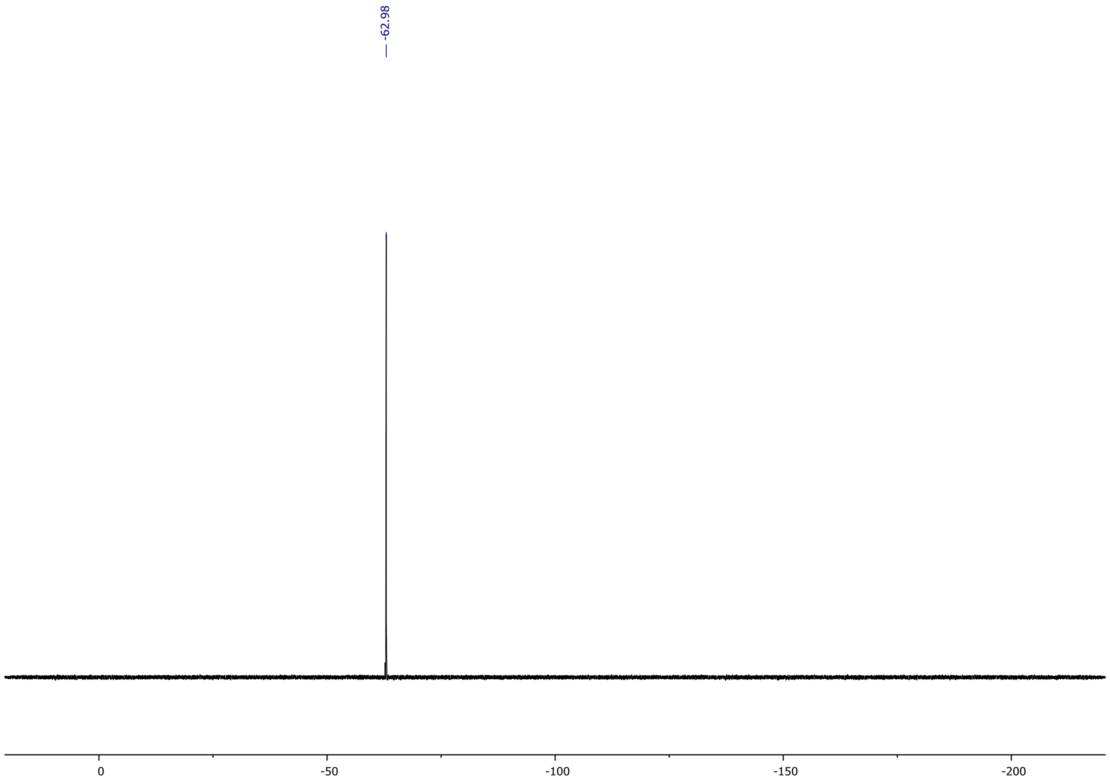

Figure S27. ^{19}F NMR spectrum of compound **1**-$ZnCl_2$ in $CDCl_3$.

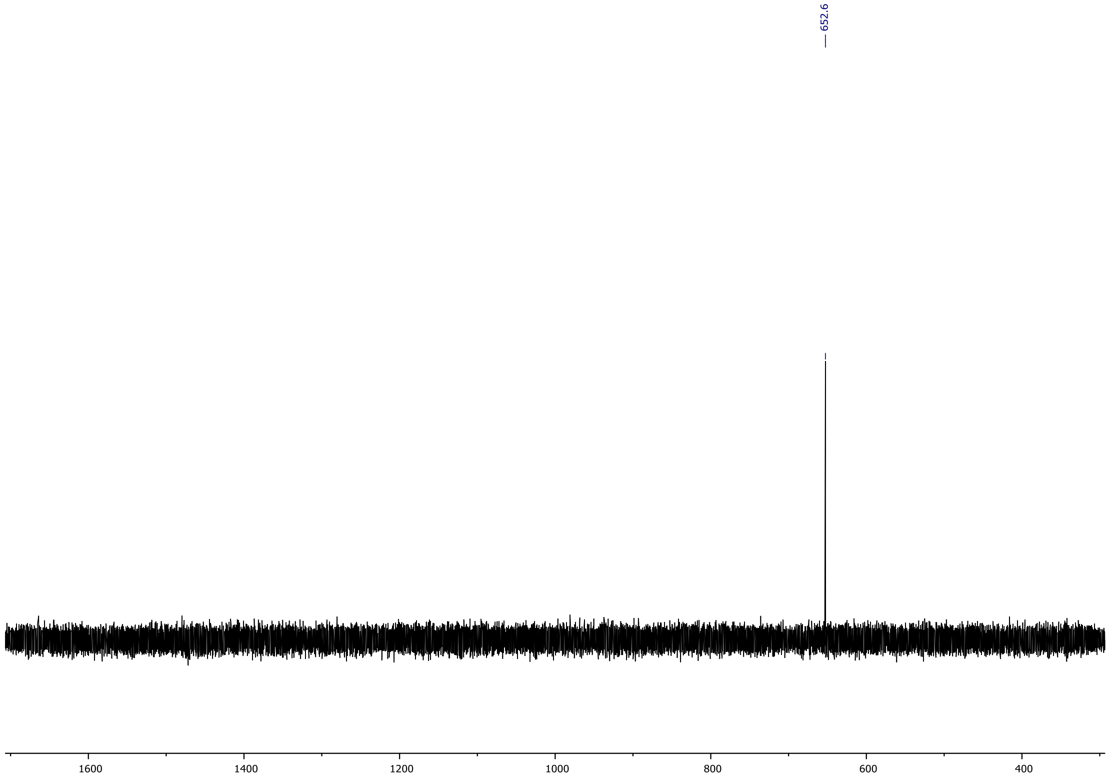

Figure S28. $^{125}Te\{^1H\}$ NMR spectrum of compound **1**-$ZnCl_2$ in CD_2Cl_2.

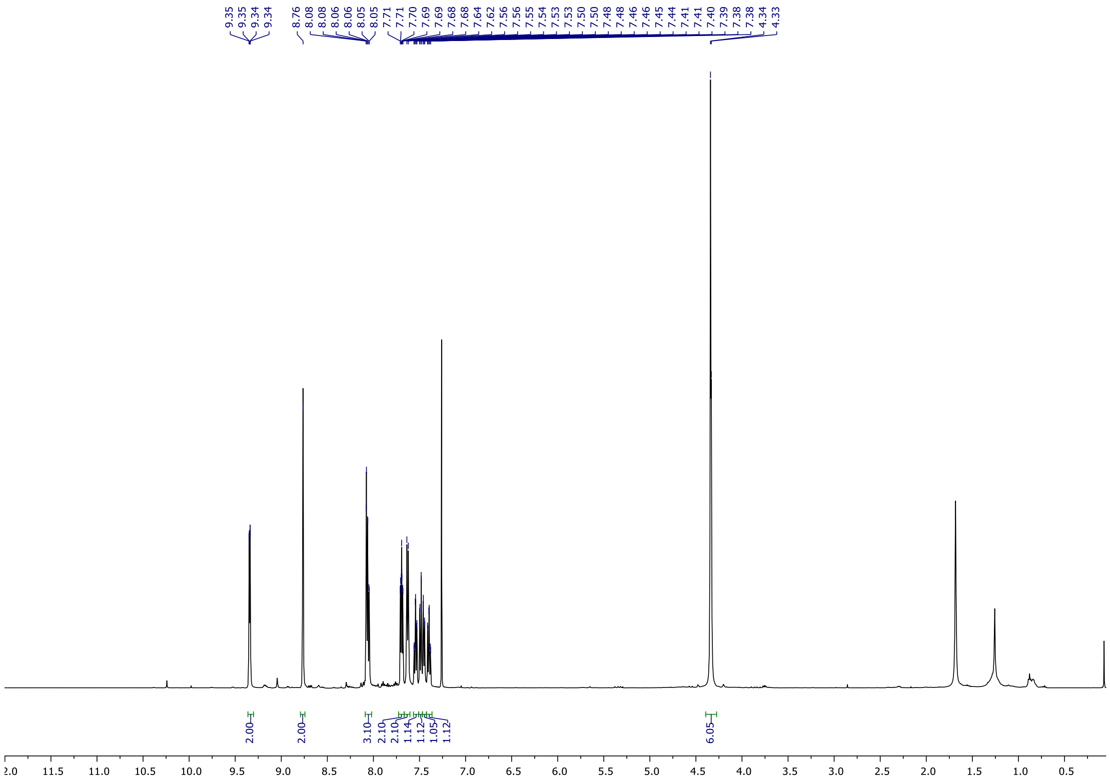

Figure S29. ^{1}H NMR spectrum of compound **2**-$ZnBr_2$ in $CDCl_3$.

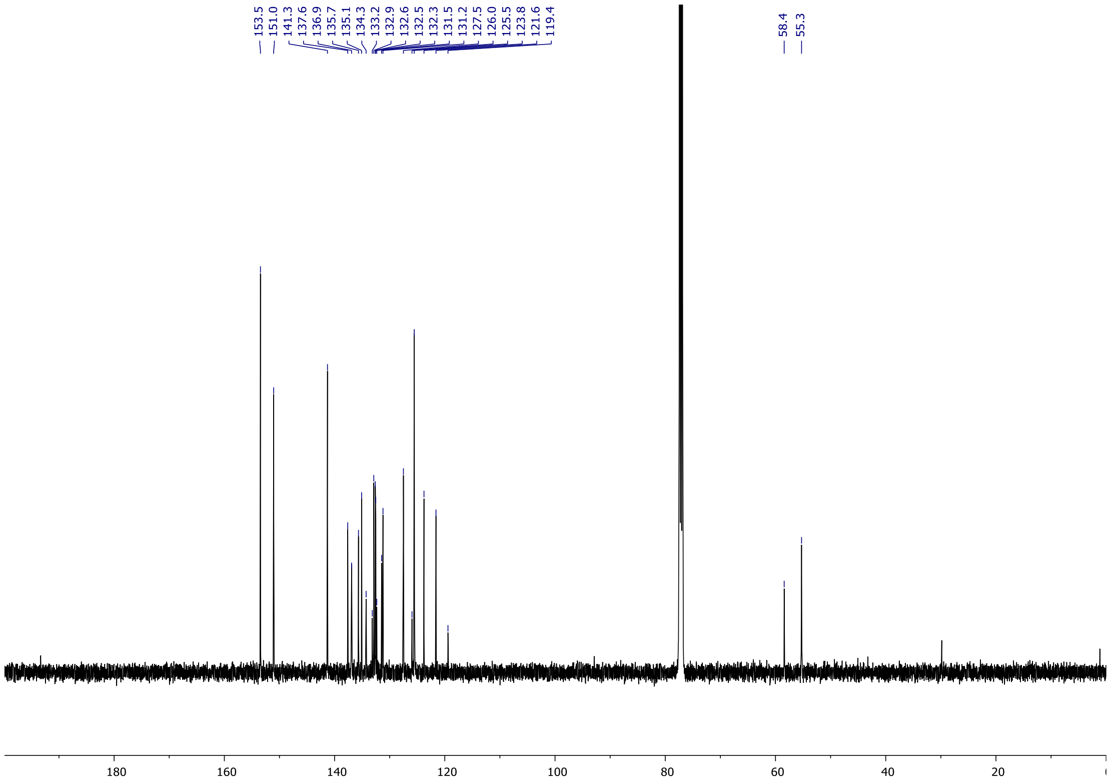

Figure S30. $^{13}C\{^{1}H\}$ NMR spectrum of compound **2**-$ZnBr_2$ in $CDCl_3$.

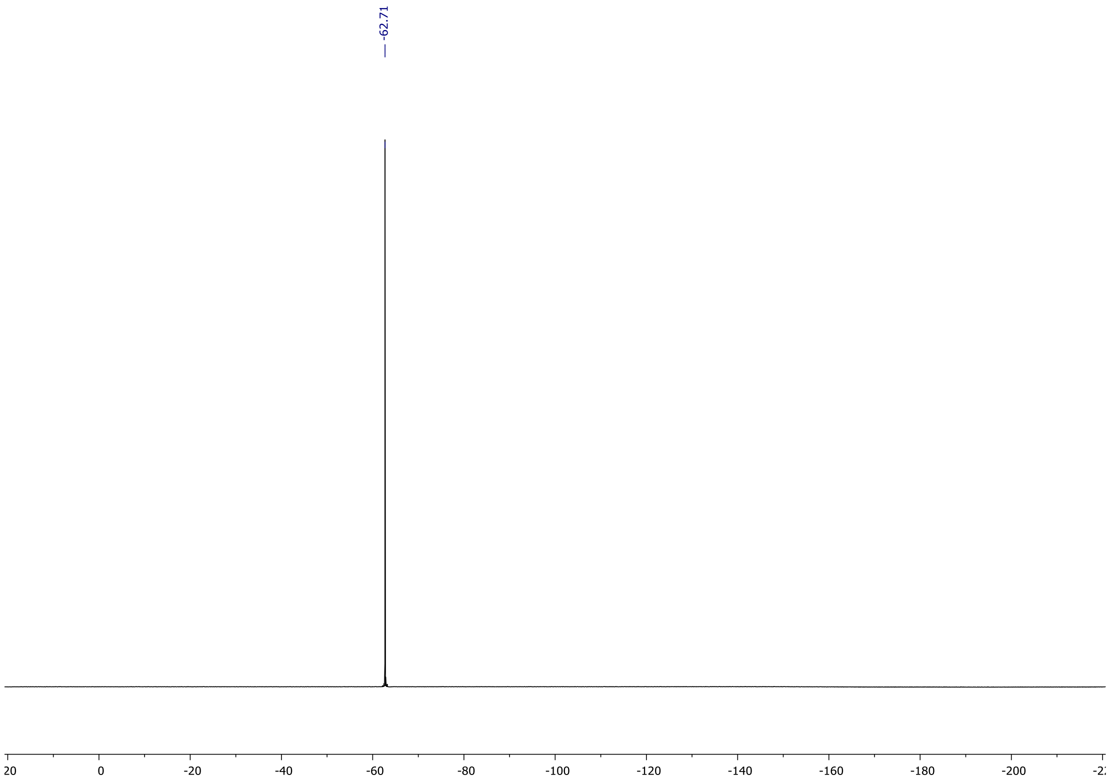

Figure S31. ^{19}F NMR spectrum of compound **2**-$ZnBr_2$ in $CDCl_3$.

2 Zinc Ion Binding Studies

UV-vis absorbance spectra were recorded on a Shimadzu UV-2502PC UV-vis spectrophotometer. In a typical experiment, a 40 mM stock solution of $ZnSO_4$ in H_2O was added by small increments (3 µL) to a 1 cm quartz cuvette containing a 1:1 DMSO/MeOH solution (3 mL) of the tellurium derivative (0.4 mM). After stirring for 1 min, the UV-vis spectrum was recorded. The changes in absorbance at selected wavelengths were used to plot binding isotherms which were fitted to the following equation:

$$\Delta I = \frac{\varepsilon_\Delta}{2(V_0+V_{Zn})}\left\{\left(c_0V_0 + c_{Zn}V_{Zn} + \frac{V_0+V_{Zn}}{K_a}\right) - \sqrt{\left(c_0V_0 + c_{Zn}V_{Zn} + \frac{V_0+V_{Zn}}{K_a}\right)^2 - 4c_0V_0c_{Zn}V_{Zn}}\right\} \quad \text{(S1)}$$

with:

c_0 = concentration of the host solution

V_0 = volume of the host solution

c_{Zn} = concentration of the $ZnSO_4$ stock solution

V_{Zn} = volume of the $ZnSO_4$ stock solution

K_a = binding constant

$\varepsilon_\Delta = \varepsilon - \varepsilon_0$, where ε is the relative molar absorptivity of the complex and ε_0 is the relative molar absorptivity of the ligand

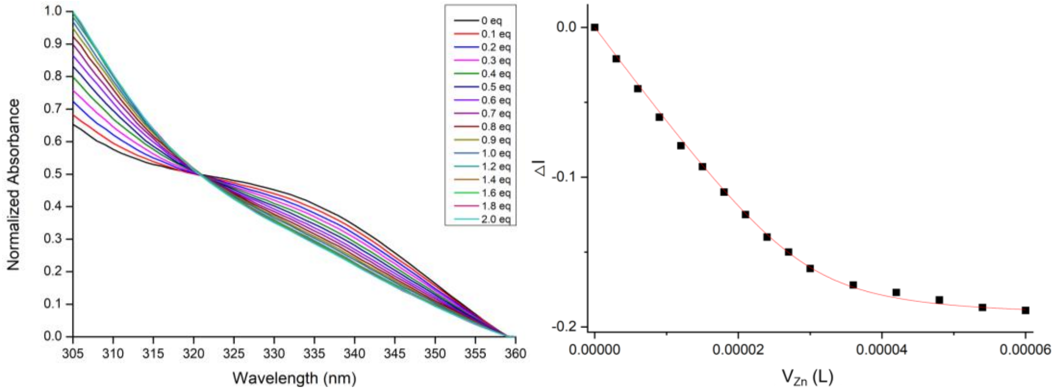

Figure S32. Changes observed in the UV-vis spectrum of a 0.4 mM solution of compound **1** upon incremental addition of $ZnSO_4$. Left: Full spectra. Right: Absorption changes at 330 nm fitted to a 1:1 binding isotherm based on equation S1 with $K_a = 5.4 \pm 1.0 \times 10^4$ M^{-1} ($\varepsilon_\Delta = -502.3 \pm 7.6$ $M^{-1}\cdot cm^{-1}$, $R^2 = 0.9987$).

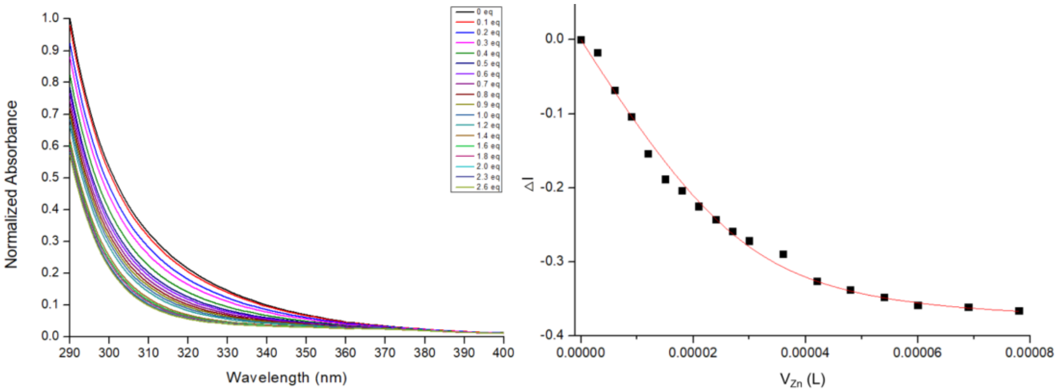

Figure S33. Changes observed in the UV-vis spectrum of a 0.4 mM solution of compound **1** upon incremental addition of $ZnSO_4$. Left: Full spectra. Right: Absorption changes at 295 nm fitted to a 1:1 binding isotherm based on equation S1 with $K_a = 1.9 \pm 0.5 \times 10^4$ M^{-1} ($\varepsilon_\Delta = -1018 \pm 35$ $M^{-1}\cdot cm^{-1}$, $R^2 = 0.9902$).

3 Reduction Studies

Reduction of compound **2** to compound **1** using excess GSH was monitored by ^{1}H NMR in DMSO-d_6. Due to the low solubility of **2** in DMSO, the spectrum of **2** was recorded at 50 °C. The reduction was carried out at 25 °C. The full spectra and comparisons are provided below.

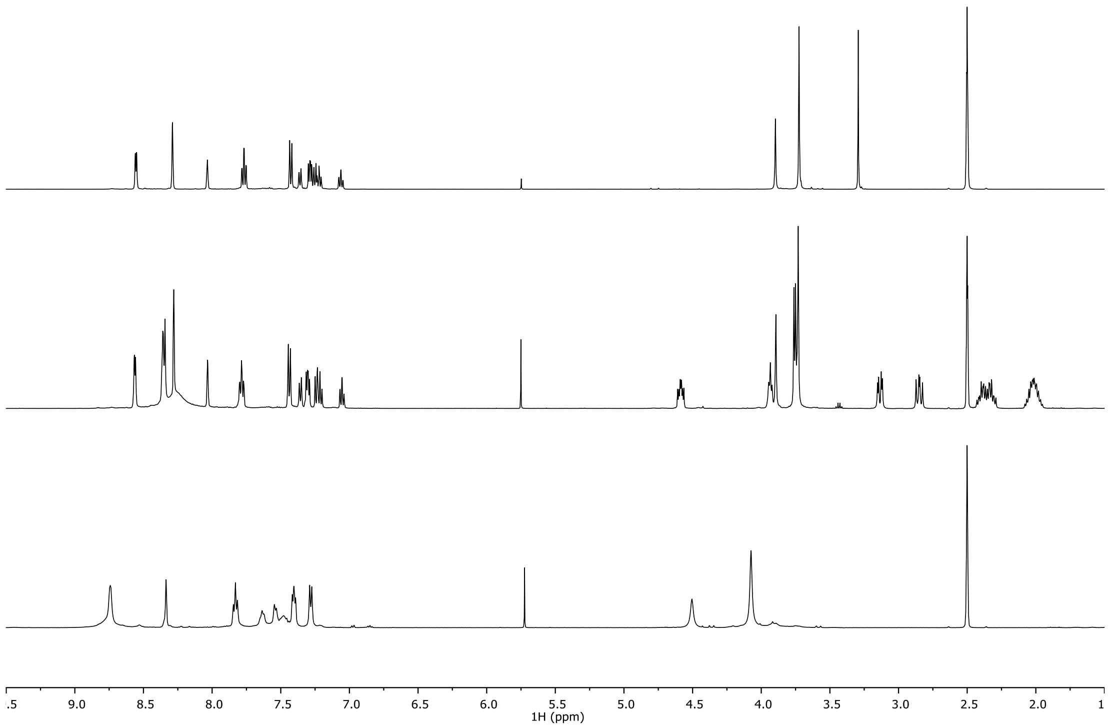

Figure S34. ^{1}H NMR spectra of **1** (top, 25 °C), **2** (bottom, 50 °C) and **2** with excess GSH (middle, 25 °C). All spectra are recorded in DMSO-d_6.

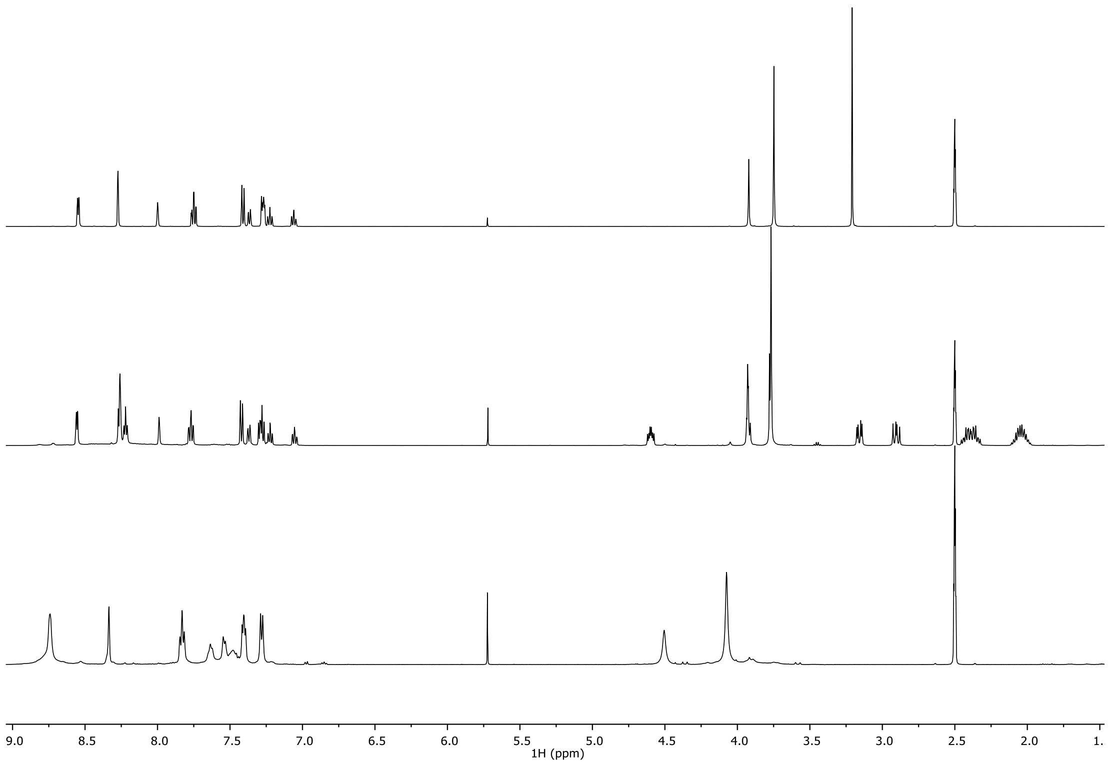

Figure S35. ^{1}H NMR spectra of **1** (top, 50 °C), **2** (bottom, 50 °C) and **2** with excess GSH (middle, 50 °C). All spectra are recorded in DMSO-d_6.

4 Transport Studies

4.1 Vesicles Preparation

80:20 EYPC/cholesterol loaded with Magnesium Green (MgG). The vesicles were prepared according to a previously established method.[4-6] A thin film of lipids was prepared by evaporating a solution of EYPC (30 mg) and cholesterol (4 mg) in 8:2 ratio dissolved in $CHCl_3$ (1.0 mL). The film was dried under vacuum overnight. A solution of MgG (1 mL, 100 mM NaCl, 10 mM HEPES, 50 μM MgG, and 100 μM EDTA, pH = 7.0) was added to suspend the lipid film. The suspension was subjected to 9 freeze-thaw (liquid N_2 bath, 49 °C water bath) cycles and then extruded through a 200 nm polycarbonate membrane 33 times. After extrusion, the extravesicular component was removed through a size exclusion column (loaded with Sephadex G-50) using an external buffer solution (100 mM NaCl, 10 mM HEPES, and 100 μM EDTA, pH = 7.0) as the eluent. These vesicles could be stored in a fridge at 4–6 °C and used within 3 d.

70:30 POPC/cholesterol loaded with lucigenin. The vesicles were prepared according to a previously established method.[4, 7] A thin film of lipids was prepared by evaporating a solution of POPC (40 mg) and cholesterol (9 mg) in 7:3 ratio dissolved in $CHCl_3$ (1.6 mL) . The film was dried under vacuum overnight. A solution of lucigenin (1 mL, 112.5 mM Na_2SO_4, and 0.8 mM lucigenin) was added to suspend the lipid film. The suspension was subjected to 9 freeze-thaw (liquid N_2 bath, 49 °C water bath) cycles and then extruded through a 200 nm polycarbonate membrane 33 times. After extrusion, the extravesicular component was removed through a size exclusion column (loaded with Sephadex G-50) using an external buffer solution (112.5 mM Na_2SO_4) as the eluent. These vesicles were used within 5 h of preparation to ensure stability and reproducibility of the experiments.

4.2 Zinc Ion Transport Activity Studies

Zinc transport was studied using an MgG assay that was adapted from recent literature reports,[5-6] using a fluorimeter equipped with magnetic stirring, an injection port, and a cell holder. The samples were excited at 506 nm, and the emission monitored at 531 nm using a time resolution of 1 datapoint per second. Vesicles containing MgG were suspended in an external buffer, affording a 2 mL solution with a lipid concentration of 0.5 mM. Before the start of the experiment, a transporter solution (0.5 mM in DMSO, 2 μL, 0.1 mol% with respect to lipid concentration) was injected and stirred for 2 min to ensure proper incorporation into the liposomal membranes. Fluorescence recording was then initiated. The start time of fluorescence recording was defined as $t = 0$. At $t = 30$ s, a pulse of $ZnCl_2$ (50 mM, 40 μL) was administered to the liposomes via a micro syringe to create a 1 mM $ZnCl_2$ gradient. The data was recorded for 420 s after the addition of the $ZnCl_2$ pulse. At $t = 450$ s, Triton X-100 (5 % w/w in water, 50 μL) was added to lyse the liposomes. Data recording was continued for another 50 s, during which time the emission intensity reached

a stable level. A final reading was recorded at t = 500 s and assigned a value of 100% zinc ion influx. Every experiment was carried out 3 times to ensure reproducibility.

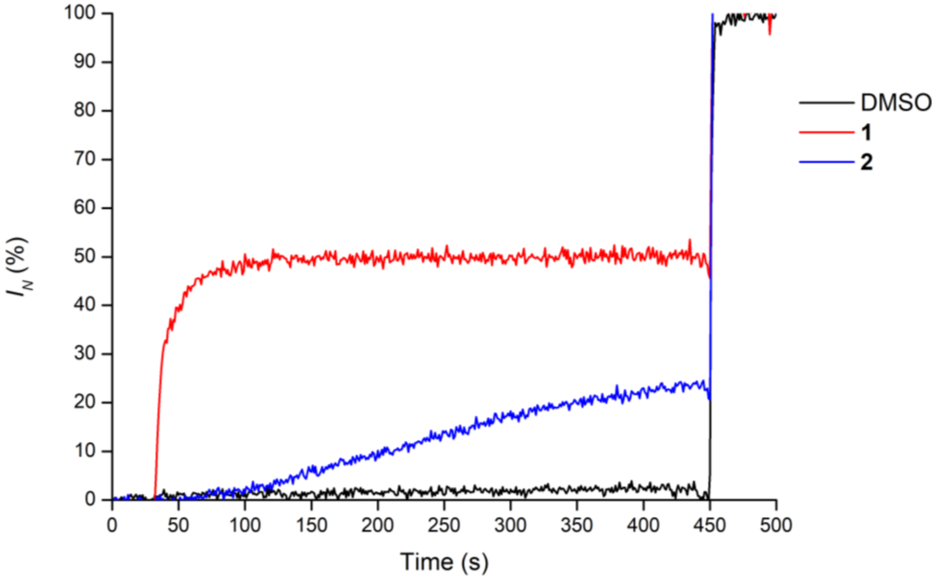

Figure S36. Zinc influx into EYPC/cholesterol vesicles mediated by the addition of a DMSO solution of transporters **1** and **2** (2 µL, 0.1 mol% with respect to lipid concentration) and the background test of DMSO.

4.3 Hill Analyses of the Zinc Transport Activity

Concentration-dependent studies were carried out for compounds **1** and **2** by using the MgG assay. The zinc influx percentage at t = 400 s was recorded (Y) and plotted versus the transporter concentration (c) according to the following Hill equation:

$$Y = Y_0 + (Y_m - Y_0)\frac{c^n}{c^n + EC_{50}{}^n} \quad \text{(S2)}$$

where Y_m = maximum zinc efflux; Y_0 = zinc efflux at t = 400 s without transporters; EC_{50} = effective concentration of the transporter; n = Hill coefficient.

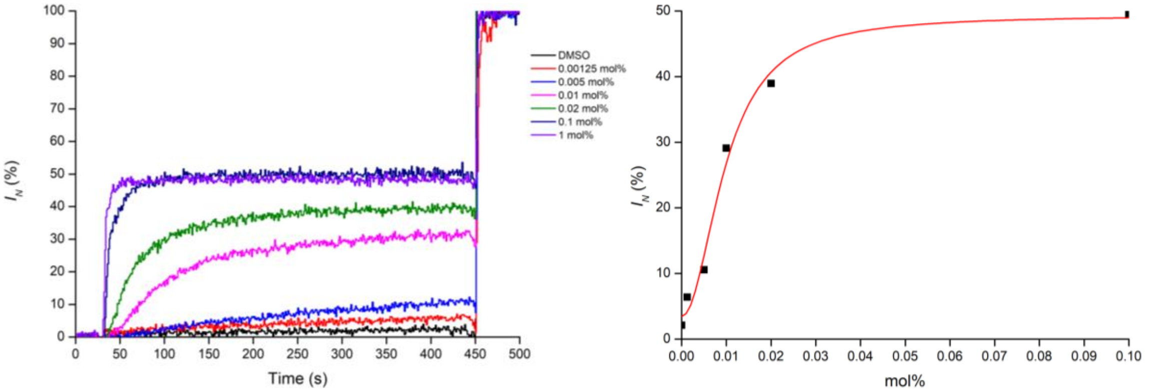

Figure S37. Left: Zinc influx into EYPC/cholesterol vesicles mediated by the addition of a DMSO solution of **1** (0.00125 mol%, 0.005 mol%, 0.01 mol%, 0.02 mol %, 0.1 mol% and 1 mol% with respect to lipid concentration). The transporter solution concentration was adjusted so that the total volume added was 2 μL. Right: Hill analysis: fitting of the zinc influx at t = 400 s versus the concentration of the transporter using equation S2. The fitting afforded EC_{50} = 0.00981 ± 0.00146 mol%, n = 2.05 ± 0.56, R^2 = 0.9734.

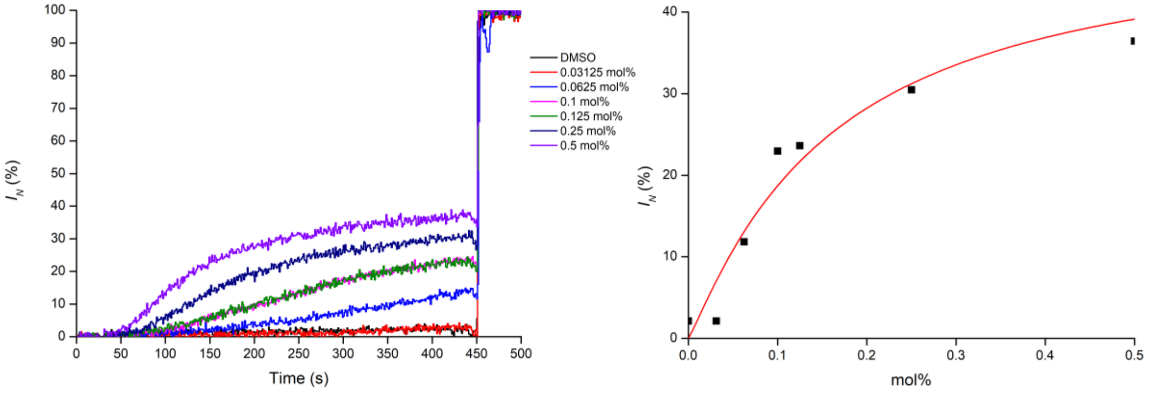

Figure S38. Left: Zinc influx into EYPC/cholesterol vesicles mediated by the addition of a DMSO solution of **2** (0.03125 mol%, 0.0625 mol%, 0.1 mol%, 0.125 mol %, 0.25 mol% and 0.5 mol% with respect to lipid concentration). The transporter solution concentration was adjusted so that the total volume added was 2 μL. Right: Hill analysis: fitting of the zinc efflux at t = 400 s versus the concentration of the transporter using equation S2. The fitting afforded EC_{50} = 0.158 ± 0.020 mol%, n = 1.12 ± 0.19, R^2 = 0.9344.

4.4 Reduction-Responsive Transport Studies Using the MgG Assay

These experiments were carried out as per the protocol described in section 4.2, with the only difference that GSH was introduced in the liposomal solution containing **2** at t = 100 s.

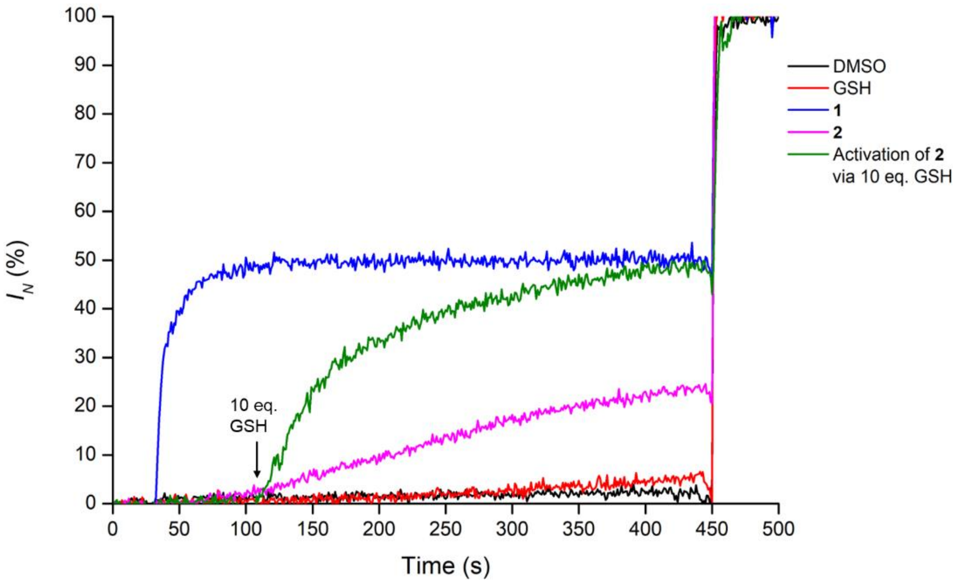

Figure S39. Zinc influx into EYPC/cholesterol vesicles mediated by the addition of a DMSO solution of transporters **1** and **2** (2 µL, 0.1 mol% with respect to lipid concentration). The background response induced by pure DMSO and a water solution of GSH, respectively, are also shown. The activation experiment was performed by introducing a GSH solution (2 µL, 1 mol% with respect to lipid concentration) at t = 100 s into a solution containing **2** and the liposomes.

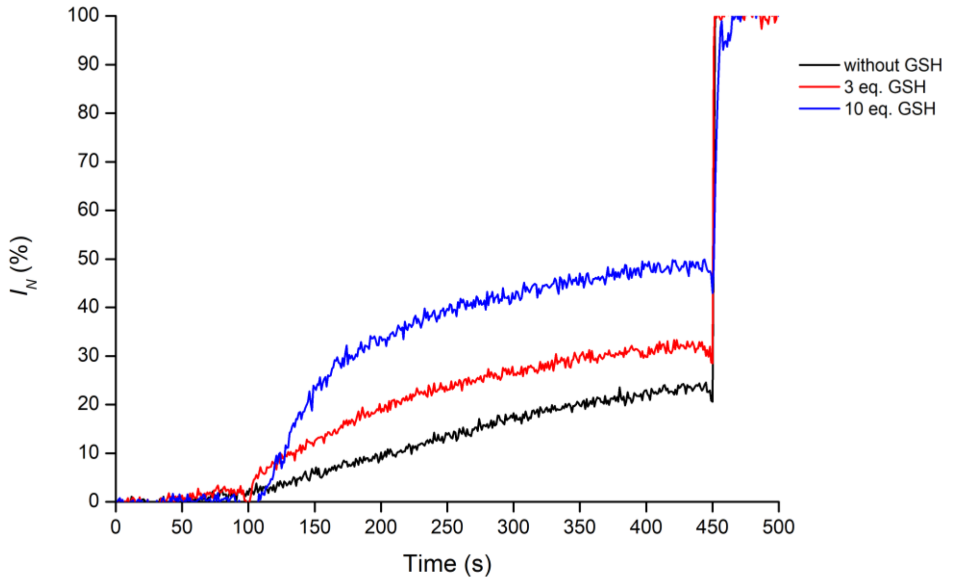

Figure S40. Comparison of zinc influx into EYPC/cholesterol vesicles mediated by the addition of a DMSO solution of **2** (2 µL, 0.1 mol% with respect to lipid concentration) in the presence or absence of GSH. A solution of GSH (3 eq or 10 eq. with respect to the transporter concentration) was added at t = 100 s. The GSH solution concentration was adjusted so that the total volume added was 2 µL.

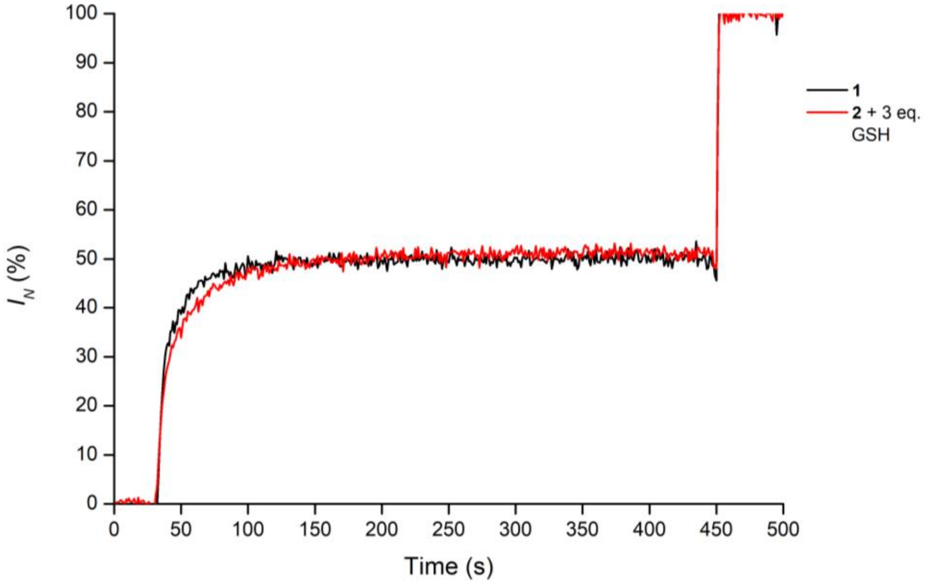

Figure S41. Comparison of zinc influx into EYPC/cholesterol vesicles mediated by **1** and **2**/GSH. In these experiments, the transporters (2 μL, 0.1 mol% with respect to lipid concentration), and GSH (2 μL, 0.3 mol% with respect to lipid concentration) in the case of **2**, were added at t = -200 s.

4.5 Pre-Incorporation Transport Studies Using the MgG Assay

Compounds **1** and **2** were respectively preincorporated into vesicles by repeating the protocol described in section 4.1 at the only difference that the transporters (0.1 mol% with respect to the lipid concentration) was added to the initial $CHCl_3$ containing the lipid and cholesterol. The reaction was stirred for 1 min before solvent evaporation. The transport studies with preincorporated compounds **1** and **2** were monitored using the MgG assay as described in section 4.2. In another experiment, vesicles with **2** preincorporated in the membranes were treated with GSH (2 μL, 1.0 mol% with respect to the lipid concentration) and stirred for 2.5 min before transport was initiated by administration of a $ZnCl_2$ pulse as described in section 4.2.

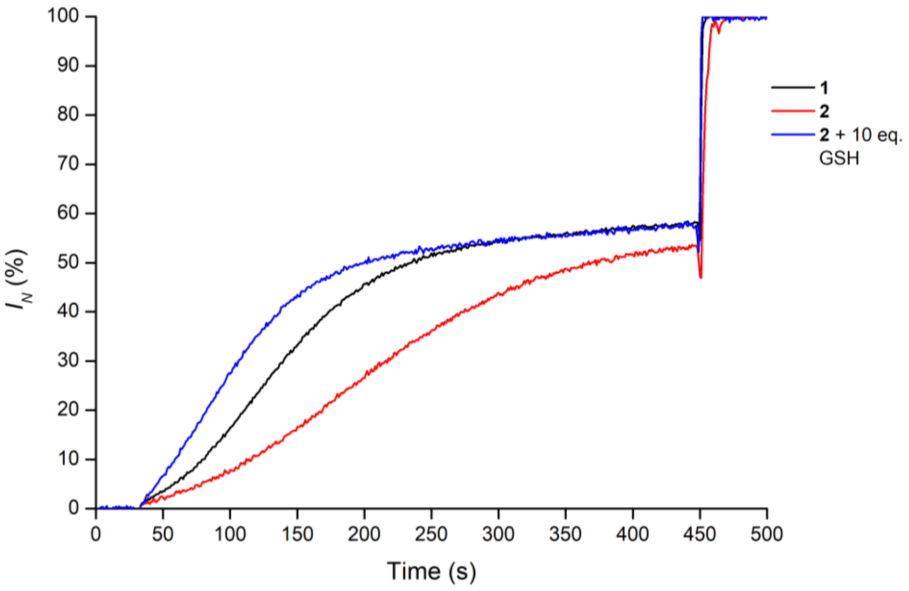

Figure S42. Zinc influx into EYPC/cholesterol vesicles prepared with preincorporation of **1** or **2** (0.1 mol% with respect to lipid concentration). The influx profile obtained after the addition GSH (10 eq.) to the vesicles containing preincorporated **2** is also shown.

4.6 Chloride Ion Transport Activity Studies

Chloride anion transport was studied using a lucigenin-based assay that was adapted from literature reports.[7-8] This assay was carried out using a fluorimeter equipped with magnetic stirring, an injection port, and a cell holder. The samples were excited at 430 nm, and the emission monitored at 505 nm using a time resolution of 1 datapoint per second. Vesicles containing lucigenin were suspended in an external buffer affording a 3 mL solution with a lipid concentration of 0.7 mM. Before the start of the experiment, a transporter solution (5 mM in methanol, 4.2 μL, 1 mol% with respect to lipid concentration) was injected and stirred for 2 min to ensure proper incorporation into the liposomal membranes. Fluorescence recording was then initiated. The start time of fluorescence recording was defined as $t = 0$. At $t = 30$ s, a pulse of 2 M $ZnCl_2$ was administered to the liposomes via a micro syringe to create a 12.5 mM $ZnCl_2$ gradient. The data was recorded for 600 s after the addition of the $ZnCl_2$ pulse. At $t = 630$ s, Triton X-100 (5 % w/w in water, 50 μL) was added to lyse the liposomes. Data recording was continued for 90 s during which time the emission intensity reached a stable level. A final reading was recorded at $t = 720$ s. Every experiment was carried out 3 times to ensure reproducibility. Due to the lipid permeable nature of lucigenin, the abrupt decrease in intensity observed upon addition of the $ZnCl_2$ pulse was not ascribed to chloride influx into the vesicles.[7] Accordingly, the fluorescence intensity at $t = 33$ s was designated as the normalized reference.

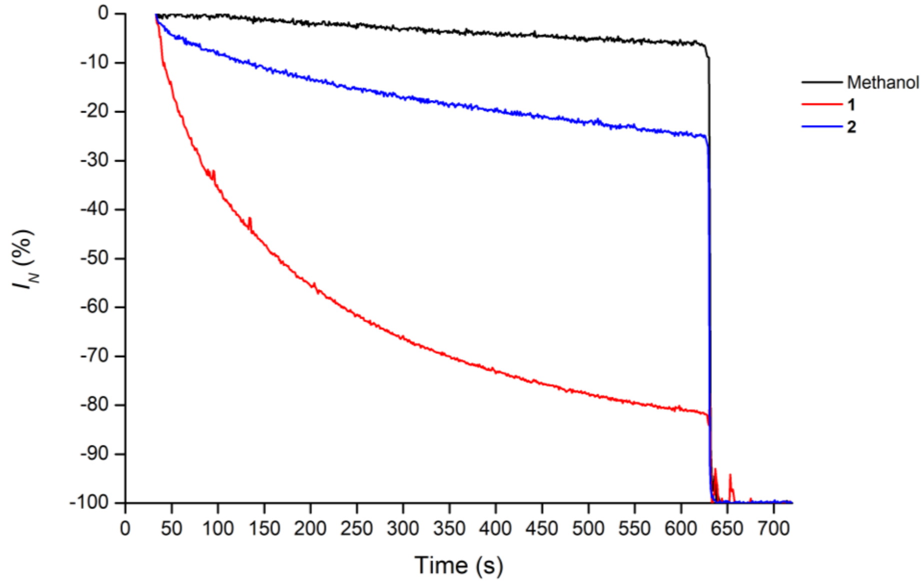

Figure S43. Chloride influx into POPC/cholesterol vesicles mediated by the addition of a methanol solution of transporters **1** and **2** (4.2 µL, 1 mol% with respect to lipid concentration). The background response in the presence of methanol is also shown.

4.7 Reduction-Responsive Transport Studies Using the Lucigenin Assay

These experiments were carried out as per the protocol described in section 4.6, with the only difference that GSH was introduced in the liposomal solution containing **2** at $t = 210$ s.

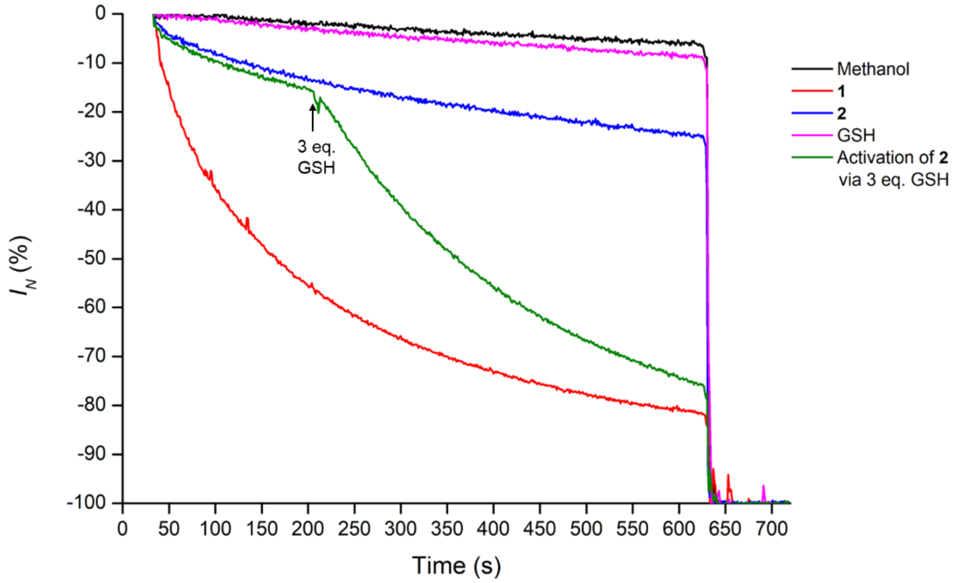

Figure S44. Chloride influx into POPC/cholesterol vesicles mediated by the addition of a methanol solution of transporters **1** and **2** (4.2 µL, 1 mol% with respect to lipid concentration). The background responses induced by pure methanol and a water solution of GSH, respectively, are also shown. The activation experiment was performed by introducing a GSH solution (4.2 µL, 3 mol% with respect to lipid concentration) at $t = 210$ s into a solution containing **2** and the liposomes.

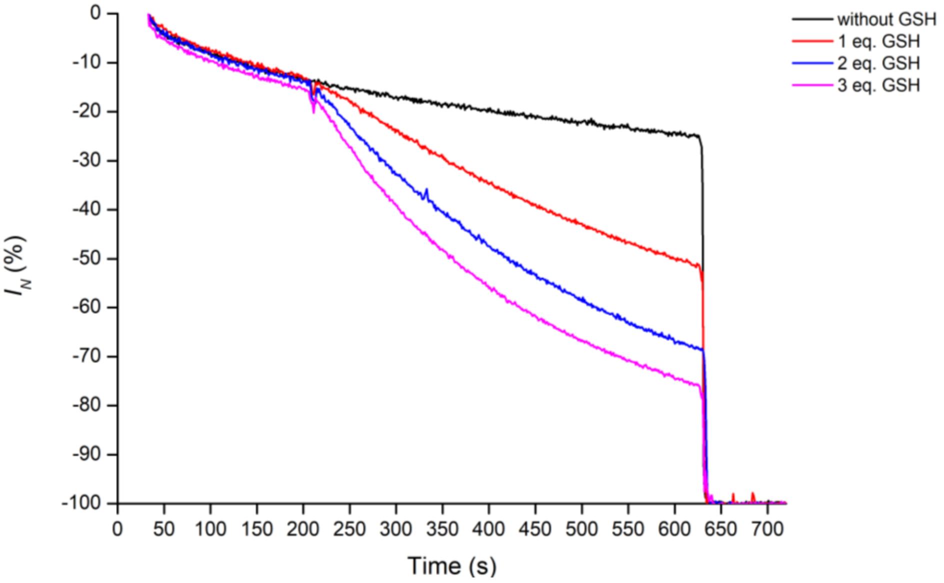

Figure S45. Comparison of chloride influx into POPC/cholesterol vesicles mediated by the addition of a methanol solution of **2** (4.2 µL, 1 mol% with respect to lipid concentration) in the presence or absence of GSH. A solution of GSH (1 eq., 2 eq. or 3 eq. with respect to the transporter concentration) was added at t = 210 s. The GSH solution concentration was adjusted so that the total volume added was 4.2 µL.

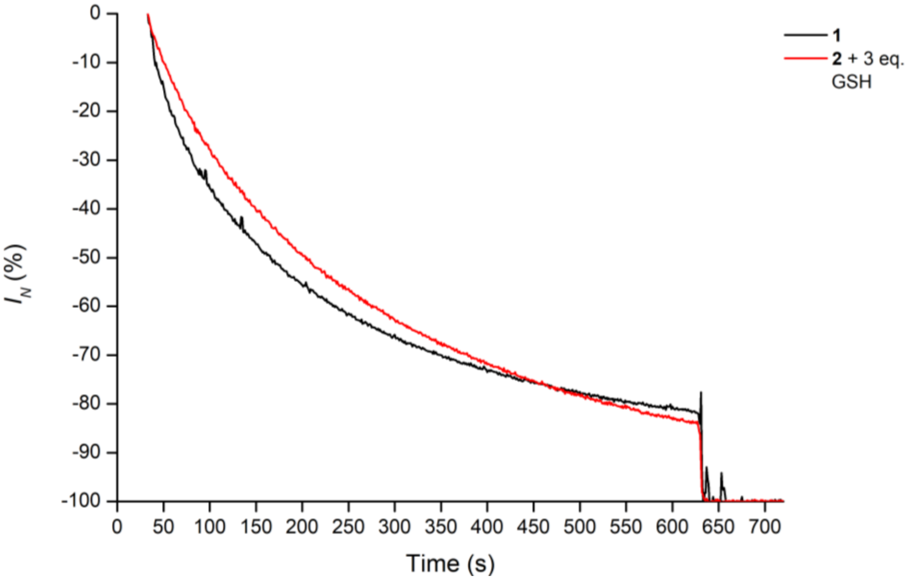

Figure S46. Comparison of chloride influx into POPC/cholesterol vesicles mediated by by **1** and **2**/GS. . In these experiments, the transporters (4.2 µL, 1 mol% with respect to lipid concentration), and GSH (4.2 µL, 3 mol% with respect to lipid concentration) in the case of **2**, were added at t = -200 s.

5 X-ray Crystallographic Data

The crystallographic measurements were performed at 110 K using a Bruker D8 Quest (Mo source) diffractometer equipped with Photon III detectors or a Synergy Rigaku (Mo source) diffractometer equipped with a HyPix-6000HE detector. Semi-empirical absorption corrections were applied using the Bruker SADABS software package.[9] The structures were solved by direct methods with SHELXT to locate all non-hydrogen atoms.[10] Subsequent refinement using a difference Fourier map against F^2 with the SHEXL package allowed for the location of the remaining non-hydrogen atoms, which were refined anisotropically. H atoms were added in calculated positions using a riding model. Structure refinements were performed using Olex2.[11] CCDC 2499458-2499461 contain the supplementary crystallographic data for this paper. These data can be obtained free of charge via www.ccdc.cam.ac.uk/data_request/cif, or by emailing data_request@ccdc.cam.ac.uk.

6 Computational Studies

6.1 General Methods

The structures of compounds **1**, **2**, **1**-$ZnCl_2$ and **2**-$ZnCl_2$ were optimized using DFT methods as implemented in Gaussian 16[12] using the B3LYP-D3(BJ)[13] functional and a def2-TZVP basis set.[14] No imaginary frequencies were found for all of the optimized structures, confirming that a local minimum on the potential energy hypersurface had been reached. Optimized structures were visualized by GaussianView 6. NBO analysis was performed using the same functional and basis set using the NBO 7.0 program.[15] The resulting NBOs were visualized using Avogadro.[16]

6.2 Optimized Structures and Coordinates of the Tellurium Compounds

Table S1. Cartesian coordinates of the optimized structure of compound **1**

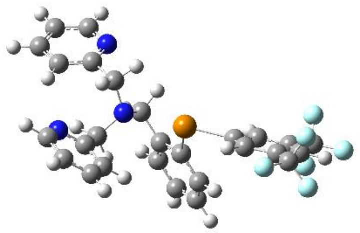

Te	0.084693	0.380574	-0.344870	C	4.375808	1.223512	-0.608673
F	4.395688	-3.342453	-1.133197	C	5.003450	-0.010102	-0.504113
F	4.566376	-3.050254	0.988296	C	4.220923	-1.137681	-0.332815
F	6.178633	-2.456131	-0.310918	C	2.835602	-1.045452	-0.276625
F	5.749934	2.468982	-2.054167	C	5.230545	2.442853	-0.819499
F	6.262958	2.473764	0.032055	C	4.848918	-2.497940	-0.199240
F	4.544984	3.577273	-0.656587	C	-2.380255	-1.637896	0.231838
N	-2.739202	-0.236476	0.017147	C	-3.345761	0.008494	-1.296243
N	-4.466429	-1.995682	-2.061524	C	-3.523395	0.295260	1.133855
N	-4.652124	2.394140	0.659080	C	-4.616832	-0.769195	-1.555714
C	-0.180442	-0.970993	1.266525	C	-5.867076	-0.233099	-1.255549
C	0.771612	-1.077323	2.275103	C	-6.996631	-1.000191	-1.493009
C	0.593320	-1.977528	3.316709	C	-6.843031	-2.272429	-2.019738
C	-0.547629	-2.764246	3.365084	C	-5.556877	-2.721672	-2.284462
C	-1.506909	-2.641400	2.370527	C	-3.555377	1.801951	1.131225
C	-1.336514	-1.754837	1.312577	C	-2.460857	2.524706	1.599960
C	2.209306	0.191293	-0.366069	C	-2.508614	3.906929	1.573000
C	2.996244	1.330456	-0.531425	C	-3.648596	4.525389	1.081066

C	-4.689692	3.725033	0.638910	H	-3.540182	1.078405	-1.378989
H	1.661507	-0.460805	2.251285	H	-3.045659	-0.046929	2.054041
H	1.344326	-2.054229	4.092202	H	-4.552386	-0.086113	1.126041
H	-0.695263	-3.463186	4.177725	H	-5.932692	0.763338	-0.835623
H	-2.407106	-3.244015	2.408270	H	-7.981696	-0.609166	-1.271790
H	2.536399	2.308876	-0.588314	H	-7.695234	-2.905451	-2.225730
H	6.081371	-0.086379	-0.549933	H	-5.395238	-3.710877	-2.699340
H	2.248287	-1.948543	-0.152941	H	-1.592433	1.998735	1.975874
H	-1.979955	-2.018935	-0.712137	H	-1.672792	4.493138	1.932793
H	-3.252246	-2.256416	0.479834	H	-3.733944	5.602554	1.042183
H	-2.611087	-0.272381	-2.052863	H	-5.598348	4.170689	0.249161

Table S2. Cartesian coordinates of the optimized structure of compound **2**

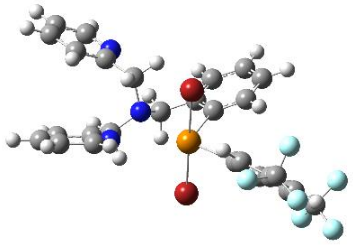

Te	8.856000	13.061000	6.822000	C	7.500000	12.967000	9.558000
Br	6.821000	12.965000	5.078000	C	7.443000	10.064000	7.076000
Br	10.887000	13.424000	8.555000	C	6.521000	10.379000	9.378000
F	9.043000	19.559000	8.538000	C	11.437000	11.755000	3.588000
F	4.100000	16.076000	7.163000	C	10.329000	9.326000	3.230000
F	10.201000	18.780000	6.944000	C	6.272000	11.140000	10.500000
N	8.744000	10.351000	6.442000	C	9.915000	9.890000	7.251000
C	7.707000	12.207000	8.409000	C	8.583000	17.356000	7.857000
F	10.550000	18.090000	8.907000	C	6.765000	12.429000	10.602000
N	10.463000	11.390000	4.423000	C	8.140000	15.050000	7.318000
N	9.247000	7.771000	8.207000	C	6.321000	16.566000	7.795000
C	9.915000	10.184000	4.226000	C	9.586000	18.447000	8.073000
C	7.242000	10.898000	8.300000	C	7.222000	17.610000	8.013000
F	4.486000	18.044000	7.847000	C	11.093000	7.739000	6.680000
F	4.467000	16.397000	9.215000	C	6.768000	15.302000	7.450000
C	8.732000	9.799000	5.081000	C	11.333000	9.732000	2.364000
C	11.896000	10.970000	2.546000	C	10.089000	8.403000	7.368000

C	11.255000	6.379000	6.875000	H	9.931000	8.468000	3.138000
C	4.843000	16.789000	8.003000	H	5.758000	10.776000	11.211000
C	9.044000	16.084000	7.527000	H	9.831000	10.267000	8.163000
C	9.406000	6.448000	8.353000	H	10.738000	10.268000	6.850000
C	10.405000	5.719000	7.727000	H	6.598000	12.943000	11.384000
H	7.905000	10.098000	4.627000	H	6.914000	18.473000	8.262000
H	8.698000	8.811000	5.143000	H	11.662000	8.212000	6.083000
H	12.584000	11.281000	1.968000	H	6.141000	14.602000	7.301000
H	7.859000	13.844000	9.624000	H	11.625000	9.164000	1.661000
H	7.403000	9.105000	7.322000	H	11.946000	5.907000	6.425000
H	6.716000	10.246000	6.431000	H	9.976000	15.924000	7.444000
H	6.197000	9.487000	9.337000	H	8.800000	5.983000	8.918000
H	11.840000	12.606000	3.717000	H	10.499000	4.787000	7.883000

Table S3. Cartesian coordinates of the optimized structure of compound **1**-$ZnCl_2$

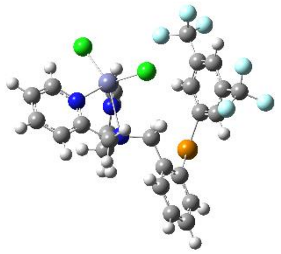

Te	2.308893	1.583682	1.756395	C	2.987261	-1.191994	-1.571428
Zn	-2.212476	-1.098693	-0.076824	C	2.566747	-2.41872	-1.080117
Cl	-0.807298	-1.700922	-1.721852	C	2.128465	-2.502284	0.233512
Cl	-3.30939	-2.788328	0.998062	C	2.086262	-1.372681	1.038951
F	1.386957	-4.722009	-0.136729	C	3.449481	-1.046807	-2.995699
F	2.648077	-4.362297	1.588875	C	1.679863	-3.819505	0.807459
F	0.593389	-3.677789	1.593896	C	1.642195	2.943326	0.246176
F	4.733987	-0.624071	-3.057652	C	2.278572	4.186947	0.24534
F	3.376745	-2.195423	-3.685557	C	1.908808	5.173315	-0.657085
F	2.715045	-0.128574	-3.662175	C	0.923037	4.904454	-1.597608
N	-1.554781	1.373741	-0.542888	C	0.305239	3.663107	-1.608882
N	-3.897408	-0.068431	-0.927654	C	0.624003	2.668653	-0.679496
N	-1.540564	-0.141994	1.737202	C	-0.101252	1.342193	-0.72774
C	2.505548	-0.145361	0.536687	C	-1.982676	2.006732	0.686718
C	2.974123	-0.057768	-0.767424	C	-1.593957	1.184086	1.892599

C	-1.332388	1.779682	3.119236	H	-0.436312	3.453197	-2.367295
C	-0.994105	0.982118	4.199606	H	0.093281	0.828068	-1.666641
C	-0.916384	-0.392466	4.022569	H	0.292614	0.689772	0.041734
C	-1.208356	-0.917506	2.775652	H	-3.072449	2.09055	0.671025
C	-2.362981	1.668583	-1.705016	H	-1.587047	3.024089	0.802218
C	-3.750888	1.07795	-1.601081	H	-1.379145	2.8564	3.211083
C	-4.832534	1.691908	-2.219707	H	-0.780021	1.426949	5.162553
C	-6.08242	1.094722	-2.153467	H	-0.646575	-1.052768	4.834431
C	-6.221042	-0.097545	-1.457725	H	-1.209807	-1.981863	2.586522
C	-5.101877	-0.646162	-0.853646	H	-2.464052	2.741918	-1.914804
H	3.300355	0.890725	-1.171917	H	-1.868696	1.209641	-2.565175
H	2.549473	-3.288036	-1.717867	H	-4.688216	2.626651	-2.744942
H	1.687949	-1.447506	2.041146	H	-6.935401	1.556982	-2.633144
H	3.082029	4.378965	0.94438	H	-7.175953	-0.597034	-1.376555
H	2.40901	6.132849	-0.642766	H	-5.135465	-1.569828	-0.288981
H	0.648982	5.651284	-2.331282				

Table S4. Cartesian coordinates of the optimized structure of compound **2**-$ZnCl_2$

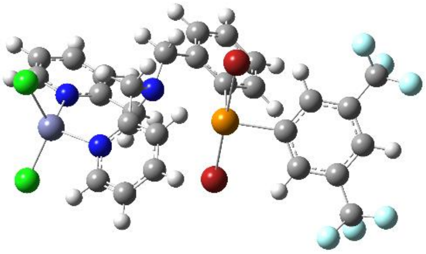

Te	0.91087	-0.15333	0.187769	C	-2.46423	0.223839	1.575698
Zn	-5.21494	-0.89503	0.205448	C	3.618096	-1.4069	-0.56662
Br	1.18474	0.792328	2.72533	C	-1.36324	2.203949	0.688516
Cl	-5.94435	-2.13527	-1.48461	C	0.81938	1.845219	-0.56258
Cl	-6.21131	-0.22262	2.080995	C	-2.46288	-1.27837	1.51408
F	4.832861	-3.84839	-1.14464	C	1.821029	2.285105	-1.41825
F	5.724467	-2.54169	-2.62576	C	-2.33805	0.592781	-0.89538
N	-1.72706	0.809428	0.435575	C	-3.56776	1.418213	-1.1725
F	6.824437	-3.04455	-0.82714	C	1.778709	3.57834	-1.92064
N	-4.74208	1.002749	-0.67569	C	5.001273	-1.55248	-0.60791
N	-3.43747	-1.87854	0.814505	C	5.264441	0.535761	0.537936
C	-0.25745	2.663917	-0.22404	C	3.884475	0.682443	0.604493
C	3.06669	-0.28797	0.041003	C	-1.46044	-2.02099	2.126029

C	-5.83763	1.749229	-0.85714	H	-1.01329	2.262787	1.71973
C	0.728447	4.418477	-1.57422	H	-2.21997	2.880461	0.595547
C	5.598258	-2.75144	-1.29798	H	2.626329	1.626247	-1.70693
C	-3.47787	2.599754	-1.90076	H	-2.56564	-0.46445	-1.00075
C	-3.42805	-3.20855	0.663804	H	-1.58566	0.826975	-1.64519
C	5.829785	-0.58083	-0.06504	H	2.56083	3.920574	-2.58482
C	-0.28241	3.956735	-0.74309	H	3.459982	1.541601	1.102819
C	6.147469	1.626769	1.08522	H	-0.70189	-1.51384	2.706237
C	-4.61444	3.367371	-2.10434	H	-6.74834	1.369734	-0.41302
C	-2.44737	-4.0058	1.228503	H	0.688983	5.427924	-1.96168
C	-5.81783	2.938993	-1.56416	H	-2.52101	2.903866	-2.30125
C	-1.45358	-3.40088	1.983929	H	-4.23545	-3.61678	0.069149
Br	0.657323	-1.06334	-2.33494	H	6.902777	-0.69673	-0.10052
F	5.62033	2.196812	2.182841	H	-1.11346	4.603599	-0.48972
F	6.321005	2.613387	0.17542	H	-4.56081	4.285143	-2.67528
F	7.371182	1.172822	1.409965	H	-2.47562	-5.07638	1.084523
H	-3.48976	0.598275	1.645876	H	-6.72939	3.505154	-1.69118
H	-1.93818	0.534256	2.476062	H	-0.6821	-3.99395	2.457369
H	2.987347	-2.15802	-1.02094				

6.3 Energy Calculations

Table S5. Energy data of compounds **1**, **1**-$ZnCl_2$, **2** and **2**-$ZnCl_2$

	**1**	**1**-$ZnCl_2$	**2**	**2**-$ZnCl_2$
**E (Hartree)**	-2073.8015092	-4773.8891068	-7222.2369481	-9922.3211797
**ZPE (Hartree)**	0.430277	0.435273	0.432556	0.438244
**H (Hartree)**	-2073.337644	-4773.414950	-7221.766577	-9921.839969
**G (Hartree)**	-2073.445228	-4773.531613	-7221.884057	-9921.968949

References

1. Alberto, E. E.; Muller, L. M.; Detty, M. R. Rate Accelerations of Bromination Reactions with NaBr and H2O2 via the Addition of Catalytic Quantities of Diaryl Ditellurides. *Organometallics* **2014**, *33*, 5571-5581.
2. Rosenau, C. P.; Jelier, B. J.; Gossert, A. D.; Togni, A. Exposing the Origins of Irreproducibility in Fluorine NMR Spectroscopy. *Angew. Chem. Int. Ed. Engl.* **2018**, *57*, 9528-9533.
3. Dean, P. A. W.; Manivannan, V.; Vittal, J. J. New adamantane-like mercury-chalcogen cages. 2. Synthetic and multinuclear (phosphorus-31, selenium-77, tellurium-125, mercury-199) magnetic resonance study of tellurolate-bridged mercury(II) clusters $[(m\text{-}TeR)_6(HgPR'_3)_4]^{2+}$ and $[(m\text{-}TeR)_6(Hg)(HgPR'_3)_3]^{2+}$ and related species with mixed-bridging chalcogenates. *Inorg. Chem.* **1989**, *28*, 2360-2368.
4. Clarke, H. J.; Howe, E. N.; Wu, X.; Sommer, F.; Yano, M.; Light, M. E.; Kubik, S.; Gale, P. A. Transmembrane Fluoride Transport: Direct Measurement and Selectivity Studies. *J. Am. Chem. Soc.* **2016**, *138*, 16515-16522.
5. Srimayee, S.; Prusty, B. M.; Kar, M. K.; Winterhalter, M.; Manna, D. Supramolecular Ion Channels to Engineer Zn^{2+} Ion Transport Mediated Chemical-to-Optical Signal Transduction. *Angew. Chem. Int. Ed.* **2025**, *64*, e202501634.
6. Gartland, S. A.; Johnson, T. G.; Walkley, E.; Langton, M. J. Inter-Vesicle Signal Transduction Using a Photo-Responsive Zinc Ionophore. *Angew. Chem. Int. Ed.* **2023**, *62*, e202309080.
7. Chvojka, M.; Singh, A.; Cataldo, A.; Torres-Huerta, A.; Konopka, M.; Šindelář, V.; Valkenier, H. The Lucigenin Assay: Measuring Anion Transport in Lipid Vesicles. *Anal. Sens.* **2023**, e202300044.
8. Mondal, A.; Save, S. N.; Sarkar, S.; Kuttanamkuzhi, A.; Bawari, S.; Vijayakanth, T.; Shivpuje, U.; Lahiri, M.; Mondal, J.; Sharma, S.; Talukdar, P. Pyrrole-Linked Benzimidazolyl Hydrazone Self-Assembly Forms HCl Channels and Induces Apoptosis in Cancer Cells. *Angew. Chem. Int. Ed.* **2025**, e202505019.
9. Sheldrick, G. M. *SADABS.*, University of Göttingen, Germany, 2016.
10. Sheldrick, G. M. *SHELXL-2014: Program for Crystal Structure Refinement*, University of Göttingen, Germany, 2014.
11. Dolomanov, O. V.; Bourhis, L. J.; Gildea, R. J.; Howard, J. A. K.; Puschmann, H. OLEX2: a complete structure solution, refinement and analysis program. *J. Appl. Crystallogr.* **2009**, *42*, 339-341.
12. Frisch, M. J.; Trucks, G. W.; Schlegel, H. B.; Scuseria, G. E.; Robb, M. A.; Cheeseman, J. R.; Scalmani, G.; Barone, V.; Petersson, G. A.; Nakatsuji, H.; Li, X.; Caricato, M.; Marenich, A. V.; Bloino, J.; Janesko, B. G.; Gomperts, R.; Mennucci, B.; Hratchian, H. P.; Ortiz, J. V.; Izmaylov, A. F.; Sonnenberg, J. L.; Williams; Ding, F.; Lipparini, F.; Egidi, F.; Goings, J.; Peng, B.; Petrone, A.; Henderson, T.; Ranasinghe, D.; Zakrzewski, V. G.; Gao, J.; Rega, N.; Zheng, G.; Liang, W.; Hada, M.; Ehara, M.; Toyota, K.; Fukuda, R.; Hasegawa, J.; Ishida, M.; Nakajima, T.; Honda, Y.; Kitao, O.; Nakai, H.; Vreven, T.; Throssell, K.; Montgomery Jr., J. A.; Peralta, J. E.; Ogliaro, F.; Bearpark, M. J.; Heyd, J. J.; Brothers, E. N.; Kudin, K. N.; Staroverov, V. N.; Keith, T. A.; Kobayashi, R.; Normand, J.; Raghavachari, K.; Rendell, A. P.; Burant, J. C.; Iyengar, S. S.; Tomasi, J.; Cossi, M.; Millam, J. M.; Klene, M.; Adamo, C.; Cammi, R.; Ochterski, J. W.; Martin,

R. L.; Morokuma, K.; Farkas, O.; Foresman, J. B.; Fox, D. J. *Gaussian 16 Rev. C.01*, Wallingford, CT, 2016.
13. Grimme, S.; Ehrlich, S.; Goerigk, L. Effect of the damping function in dispersion corrected density functional theory. *J. Comput. Chem.* **2011**, *32*, 1456-1465.
14. Weigend, F.; Ahlrichs, R. Balanced basis sets of split valence, triple zeta valence and quadruple zeta valence quality for H to Rn: Design and assessment of accuracy. *Phys. Chem. Chem. Phys.* **2005**, *7*, 3297-3305.
15. Glendening, E.D.; Badenhoop, J. K.; Reed, A. E.; Carpenter, J. E.; Bohmann, J. A.; Morales, C. M.; Karafiloglou, P.; Landis, C. R.; Weinhold, F. *NBO 7.0, Theoretical Chemistry Institute*, University of Wisconsin, Madison, WI, 2018.
16. Hanwell, M. D.; Curtis, D. E.; Lonie, D. C.; Vandermeersch, T.; Zurek, E.; Hutchison, G. R. Avogadro: an advanced semantic chemical editor, visualization, and analysis platform. *J. Cheminformatics* **2012**, *4*, 17.
